# Simultaneous targeting of HER family pro-survival signaling with Pan-HER antibody mixture is highly effective in TNBC: a preclinical trial with PDXs 

**DOI:** 10.1186/s13058-020-01280-z

**Published:** 2020-05-15

**Authors:** Tejaswini P. Reddy, Dong S. Choi, Ann C. Anselme, Wei Qian, Wen Chen, Johan Lantto, Ivan D. Horak, Michael Kragh, Jenny C. Chang, Roberto R. Rosato

**Affiliations:** 1 Houston Methodist Cancer Center, Houston Methodist Hospital, Houston, TX 77030 USA; 2 Texas A&M Health Science Center College of Medicine, Bryan, TX 77807 USA; 3 Symphogen A/S, Pederstrupvej 93, DK-2750 Ballerup, Denmark

**Keywords:** HER family, EGFR, Triple-negative breast cancer, HER2, HER3, PDX, Pan-HER

## Abstract

**Background:**

The human epidermal growth factor receptor (HER) family, notably EGFR, is overexpressed in most triple-negative breast cancer (TNBC) cases and provides cancer cells with compensatory signals that greatly contribute to the survival and development of resistance in response to therapy. This study investigated the effects of Pan-HER (Symphogen, Ballerup, Denmark), a novel mixture of six monoclonal antibodies directed against members of the HER family EGFR, HER2, and HER3, in a preclinical trial of TNBC patient-derived xenografts (PDXs).

**Methods:**

Fifteen low passage TNBC PDX tumor samples were transferred into the right mammary fat pad of mice for engraftment. When tumors reached an average size of 100–200 mm^3^, mice were randomized (*n* ≥ 6 per group) and treated following three 1-week cycles consisting of three times/week intraperitoneal (IP) injection of either formulation buffer (vehicle control) or Pan-HER (50 mg/kg). At the end of treatment, tumors were collected for Western blot, RNA, and immunohistochemistry analyses.

**Results:**

All 15 TNBC PDXs were responsive to Pan-HER treatment, showing significant reductions in tumor growth consistent with Pan-HER-mediated tumor downmodulation of EGFR and HER3 protein levels and significantly decreased activation of associated HER family signaling pathways AKT and ERK. Tumor regression was observed in five of the models, which corresponded to those PDX tumor models with the highest level of HER family activation.

**Conclusions:**

The marked effect of Pan-HER in numerous HER family-dependent TNBC PDX models justifies further studies of Pan-HER in TNBC clinical trials as a potential therapeutic option.

## Background

As a heterogeneous disease, breast cancer is clinically classified by taking into account the expression of estrogen receptor alpha (ERα), progesterone receptor (PR), and the presence/amplification status of the oncogenic human epidermal growth factor receptor 2 (HER2) [1]. Triple-negative breast cancer (TNBC), i.e., those not expressing ER, PR, or HER2, represent ~ 10–20% of all cases, have poorer prognosis than HER2^+^ or hormone receptor-positive tumors, and are generally characterized by an aggressive clinical course [2]. Furthermore, due to the lack of druggable targets including HER2 and ER, the main therapeutic options remain surgery and systemic chemotherapies, either individually or combined [reviewed in [3]]. Importantly, epidermal growth factor receptor (EGFR) is more often overexpressed in TNBC than in other breast cancer subtypes, making this a possible target for therapeutic intervention.

The present study investigated the effects of Pan-HER, a novel mixture of six monoclonal antibodies (mAbs) directed against members of the human epidermal growth factor receptor (HER) family EGFR/HER1, HER2, and HER3 [4], in a preclinical trial of TNBC patient-derived xenografts (PDXs). The HER family, notably EGFR, is well recognized for its pro-oncogenic activity that upon activation by corresponding ligands lead to receptor dimerization [5]. These effects are mediated through downstream signals including PI3K/AKT, RAS/RAF/MEK/ERK1/2, JAK/STAT3, and PLCγ-pathways ([5]. Several cancer types have been shown to be associated with either mutation or increased expression of the HER family members, including breast, lung, stomach, colorectal, head and neck, pancreatic carcinomas, and glioblastoma {Roskoski, 2014 #2300, [6]). Furthermore, accumulating evidence shows that the HER family is important in providing cancer cells with compensatory signals that greatly contribute to the development of resistance and survival in response to therapy [7–9]. Pan-HER was designed based on the hypothesis that simultaneous inhibition and/or downmodulation of multiple members of the HER family may result in effective disruption of tumor growth, preventing the HER-driven cell proliferation, survival, angiogenesis, and invasion [10]. Earlier studies, including clinical trials [11, 12], explore the concept of targeting simultaneously either HER family members or combining antibody-based therapy with kinase inhibitors [13, 14] and have set the stage for further investigation of this mechanistic-related concept. In this context, the potential therapeutic advantage of Pan-HER may reside in its ability to simultaneously target all three receptor tyrosine kinases (RTKs), which may in turn block, or at least significantly delay, the appearance of survival and escape mechanisms [4, 15].

The effect of Pan-HER has been investigated in a number of cell lines and xenografts representing a diverse number of cancer types including head and neck, lung [16, 17], HER2^+^ breast [18], and other malignancies shown to have a dependency on one or more of the targets, i.e., EGFR, HER2, or HER3 [4]. Pan-HER demonstrated stronger activity than single mAbs directed against single HER family members and was capable of overcoming acquired resistance due to increased ligand expression [4]. Here, we present the results of a preclinical trial of Pan-HER performed in 15 TNBC PDX models. Our results show significant antitumor activity by Pan-HER in all PDX tumor models evaluated, with a noticeable therapeutic response in those TNBC tumors whose dependency on the HER family appeared to be most pronounced.

## Methods

### Reagents

Pan-HER, a mixture of six monoclonal antibodies directed against each of the HER family members EGFR, HER2, and HER3, was generously provided by Symphogen A/S (Denmark) [4, 15, 19]. Pan-HER is formed by the combination of three sets of two antibodies each targeting non-overlapping epitopes of EGFR, HER2, and HER3.

### Mice

All protocols involving mice followed standard regulations and were approved by the Houston Methodist Research Institute Institutional Animal Care and Use Committee (IACUC). Female immunodeficient SCID/beige mice (Envigo, Indianapolis, IN) were used as the recipient strain to engraft PDXs. PDXs were originally derived by transplantation of a fresh treatment-naïve patient breast tumor biopsy into the cleared mammary gland fat pad of immunocompromised mice [20]. Low passage TNBC PDX tumor samples (2 × 2 × 2 mm; Additional file **1**: Table S1) were transferred into the right mammary fat pad of mice for engraftment. Mouse body weights were recorded as an indication of the animals’ health status; tumor volumes were measured and calculated twice weekly following the formula [0.5 × (long dimension) × (short dimension)^2^]. When tumors reached an average size of 100 to 200 mm^3^, mice were randomized (*n* ≥ 6 per group) and used to determine the response to treatment. Tumor volume fold change was calculated based on the baseline tumor volumes for each arm.

### Gene expression and data analyses

Relative expression levels or genetic alterations/modifications corresponding to HER family members (EGFR and HER3) or associated signaling pathway (i.e., focal adhesion kinase [FAK] and phosphatase and tensin homolog [PTEN]) in human breast cancer including triple-negative breast cancer, were investigated by Oncomine Cancer Microarray database analysis [21] of The Cancer Genome Atlas (TCGA); cBioPortal (Memorial Sloan Kettering Cancer Center’s Computational Biology Center, New York, NY) and NCI-GDC Data Portal database were used to interrogate for the incidence of key genes/pathways related to the present study (*n* = 8824 patients, 9052 samples in 12 studies). Microarrays were performed using Affymetrix GeneChip U133plus 2.0. Normalization and evaluation of the data were performed as previously described [22]. The array data were evaluated using the commercial software suite, Partek Genomics Suite. Specifically, data were normalized by using the RMA (robust multichip averaging) method. Gene expression levels were analyzed on a logarithmic scale. ANOVA was used to identify differentially expressed genes. Genes with a *P* value of less than 0.05 in each comparison were selected for further functional and pathway analyses by Ingenuity Pathway Analysis (IPA; Qiagen, Germantown, MD) tools. Patient survival analysis was obtained by using Kaplan-Meier analysis tools as previously described [23, 24]. EGFR and NF-κB signaling pathway-focused real-time RT-PCR analyses were performed by using Pathway PCR Arrays (RealTimePrimers, Elkins Park, PA) and the SensiFAST SYBR No-ROX One-Step Kit (Bioline USA, Taunton, MA) according to the manufacturers’ protocol. Gene expression was compared according to the *C*_T_ value. Gene expression analysis included also that performed by Ingenuity Pathway Analysis (IPA, Qiagen). The corresponding full lists of genes included in each array are described in Additional file **1**: Table S2. Each array contained 88 targeted plus 8 housekeeping gene primer sets.

### Immunohistochemistry

Immunohistochemistry assays were performed following well-established protocols as previously described [25]. After antigen retrieval (Tris-Cl, pH 9.0), paraffin-embedded sections of PDX tumors were incubated for 1 h at room temperature with the following antibodies: anti-human EGFR, clone D38B1 (Cell Signaling Technology, Danvers, MA); and anti-human HER3/ErbB3 (clone D22C5; Cell Signaling Technology).

### Western blot analysis

Analysis of proteins was performed by Western blot as previously described [25]. Briefly, 30 μg protein of whole cell lysates were subjected to SDS-PAGE electrophoresis in 4 to 20% polyacrylamide gels (Bio-Rad, Hercules, CA, USA). Proteins were transferred onto nitrocellulose membranes (Bio-Rad), incubated overnight at 4 °C with primary antibodies (1:1000), followed by washes and incubation with the appropriate secondary antibodies for 1 h (1:2000). Protein bands were developed in autoradiography films (Denville Scientific Inc., South Plainfield, NJ). Antibodies used in this study were purchased from Cell Signaling Technology and included anti-EGF Receptor (D38B1), anti-HER3/ErbB3 (D22C5), anti-phospho-EGFR (Tyr1068; D7A5), anti-phospho-HER3/ErbB3 (Tyr1289; D1B5), anti-phospho-FAK (Tyr925), anti-phospho-Stat3 (Tyr705), anti-Stat3 (79D7), anti-phospho-AKT (S4737), and anti-AKT (C67E7).

### Statistical analysis

All data were analyzed using GraphPad Prism (GraphPad Software, La Jolla, CA). Data are presented as mean ± standard error of the mean. Statistical significance between two groups was analyzed by two-tailed Student’s *t* test. Experiments with more than three groups were analyzed with one-way analysis of variance (ANOVA) and Bonferroni’s post hoc test. Statistical analysis of tumor volume was assessed by two-way ANOVA and Bonferroni’s post hoc test. A *P* value of less than 0.05 was considered significant.

## Results

### Prevalence of HER family mutations in breast cancer

**Fig. 1:**
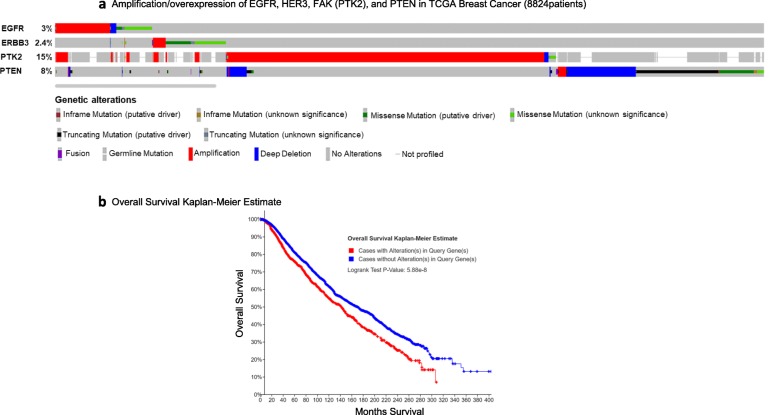
Amplification/overexpression of the HER family correlates with poor prognosis in breast cancer. **a** Data acquired from cBioPortal (NCI-GDC Data Portal) for Cancer Genomics showing the percentages of genetic modifications of different components of the HER family among breast cancer patients (8824 breast cancer patients, a total of 9052 samples from 12 studies). **b** Alterations of HER family members portends significantly poorer overall survival in TNBC patients [Kaplan-Meir analysis tools: Refs. [23, 24]]

Using public databases and analysis tools (see “Methods”) [21, 23, 24], the prevalence of mutations and/or alterations in HER family members were investigated in a pool representing 8824 breast cancer patients (a total of 9052 samples from 12 studies), including those corresponding to TNBC. Genetic alterations were found corresponding to EGFR (3%) and HER3 (2.4%) (Fig. 1a). Gene expression analysis corresponding to 13 of the PDXs used in the present study at baseline (i.e., low passage, non-treated PDX tumor tissue) was performed. Three of the PDXs including BCM-3936, BCM-4913, and MC1 displayed a very similar gene expression pattern that substantially differed from the rest of samples (Additional file 2: Figure S1a). As determined by Ingenuity Pathway Analysis (IPA) comparing these three PDXs against the rest of the models, changes in genes related to the PTEN pathway, a very well-described tumor suppressor gene [26, 27], and PTK2 (FAK) were among the top molecules (Additional file 2: Figure S1b). Both genes represent key components of the AKT/PKB survival pathway, a critical downstream signaling on HER family activation [28–31]. Based in these observations, we included into the analysis of data bases both PTK2 and PTEN, both showing also marked alterations in breast cancer samples (12% and 7%, respectively; Fig. 1a). Importantly, these mutations/alterations were directly correlated with reduced overall survival (OS; Fig. 1b). Together, these data indicate a clear association between alterations in HER family members and their corresponding signaling pathways with poorer prognosis and survival in breast cancer.

### Evaluation of Pan-HER activity against TNBC PDXs

In order to evaluate the activity of Pan-HER as a therapy for TNBC, a total of 15 different PDX tumor models were selected from our collection [20] to perform a preclinical trial. All of the PDXs used in this study were originally derived by transplantation of a fresh treatment-naïve patient breast tumor biopsy into the cleared mammary gland fat pad of immunocompromised mice [20]; they are triple negative (i.e., ER^−^, PR^−^, and HER2^−^) with positive expression of both EGFR and HER3, with the exception of BCM-4664, which displays low EGFR expression and HER3^−^ (Additional file Tables: Table S1); additional information includes, whenever available, TP53 mutations, PAM50, and Pietenpol subtype classification (Additional file Tables: Table S1) [20, 32–34]. The characterization of PDXs also included sequence analysis of tumor PDXs for mutations in *PI3KCA* (exons 9 and 20) and *EGFR* (exons 18, 19, 20, and 21) [35, 36]. No changes and/or functionally significant alterations in the mentioned exons were found (Additional file Tables: Tables S2–4; Additional file Methods).

**Fig. 2:**
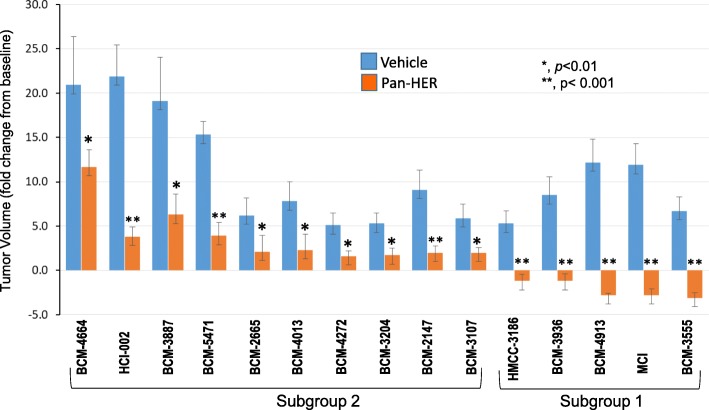
Overall responses of 15 TNBC PDX models to Pan-HER therapy. Low passage TNBC PDX tumor samples were transferred into the right mammary fat pad of mice for engraftment. Once tumors reached an average size of 100–200 mm^3^, mice were randomized (*n* ≥ 6 per group) and used to determine the response to the treatment. Regimen treatment design followed three, 1-week cycles consisting of IP injection three times/week of either formulation buffer (vehicle control) or Pan-HER (50 mg/kg). Mouse weight was recorded and tumor volumes measured and calculated, as described in “Methods”, twice weekly. Tumor volume fold change was calculated based on the baseline tumor volumes for each arm. Two-way ANOVA was used for statistical analysis

The treatment regimen followed three 1-week cycles consisting of an intraperitoneal (IP) injection three times per week of either formulation buffer (vehicle control) or Pan-HER (50 mg/kg), commencing once tumors had reached an average size of 100–200 mm^3^. The antitumor response to the Pan-HER therapy is summarized in Fig. 2. Strikingly, all 15 PDX tumor models, which had different growth rates as reflected by the fold change in tumor size within 3 weeks of starting the treatment, showed a significant reduction in tumor volume at the end of the third cycle of therapy, notably five of them which displayed tumor regression (Fig. 2). Based on these results, PDXs were divided into subgroup 1, which included those TNBC PDXs that were highly responsive (tumor regression) to treatment with Pan-HER, and the remainder, subgroup 2, that still displayed a positive response, although more moderate in terms of tumor growth inhibition (Fig. 2). Details on each of the PDX response to Pan-HER treatment are given in Additional file 2: Figures S2-S16.

**Fig. 3:**
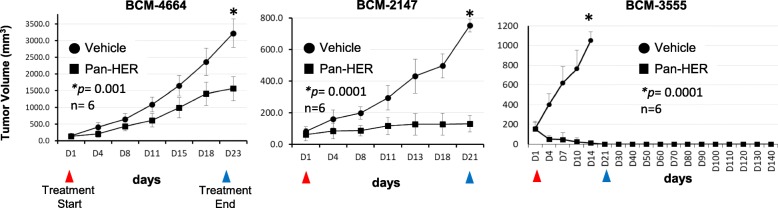
Time-course analysis of three representative TNBC PDXs. Low passage TNBC PDX tumor samples BCM-4664, BCM-2147, and BCM-3555 were transferred into the right mammary fat pad of mice for engraftment. Once tumors reached an average size of 100–200 mm^3^, mice were randomized (*n* ≥ 6 per group) and treated following the three 1-week cycle design, consisting of intraperitoneal IP injection three times/week of either formulation buffer (vehicle control) or Pan-HER (50 mg/kg). Mouse weight was recorded and tumor volumes measured and calculated, as described in “Methods”, twice weekly. Tumor volume fold change was calculated based on the baseline tumor volumes for each arm. Two-way ANOVA was used for statistical analysis. Arrows indicate the beginning (red arrow) and end (blue arrow) of treatment

The time-course response of three representative PDXs selected based on their corresponding response analysis (Fig. 2), namely BCM-4664 (fast growing, very aggressive tumor), BCM-2147 (moderate growth), and BCM-3555 (slow growth), is shown in Fig. 3. Consistent with the results shown in Fig. 2, all PDXs treated with Pan-HER displayed significant delays in tumor growth by the end of the third cycle (blue arrow; Fig. 3 and Additional file 2: Figures S2-S16). PDXs BCM-4913, MC1, and BCM-3555 (subgroup 1) were followed for an extended period (128, 58, and 145 days, respectively) to determine potential tumor recurrence. In all three cases, which were among those with the best response to the treatment, no signs of recurrence were observed (Fig. 3 and Additional file 2: Figures S14–16). Overall, the preclinical trial performed in 15 different TNBC PDXs showed that treatment with Pan-HER was highly effective at controlling tumor growth, leading in some cases to complete tumor regression and no recurrence.

### Pan-HER activity correlates with activation of the EGFR, HER3, and associated signaling pathways

**Fig. 4:**
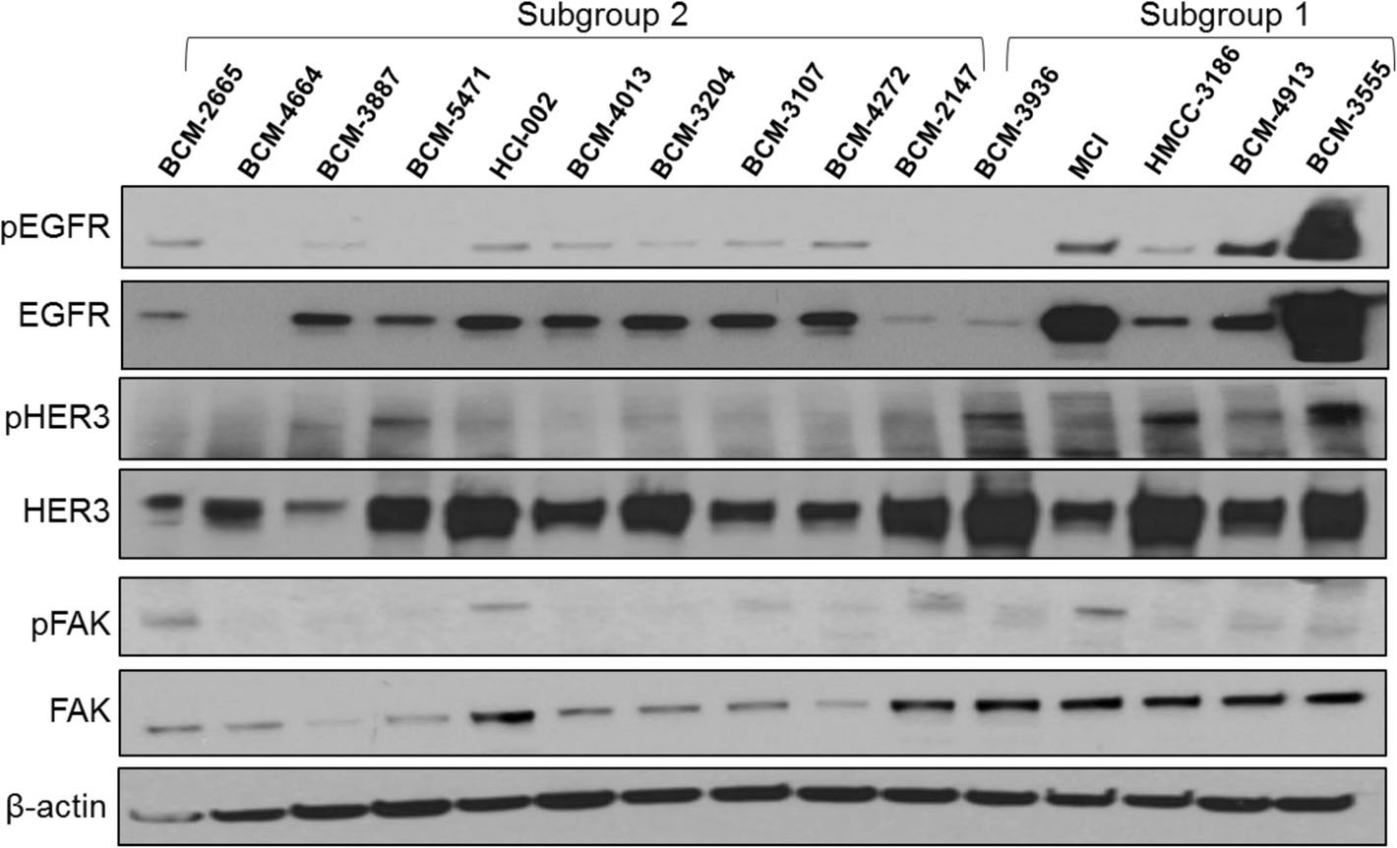
Analysis of HER family members and associated signaling pathways. Untreated PDX tumor models were analyzed by Western blot to determine the basal, initial levels of expression of HER family members EGFR and HER3, as well as associated signaling pathways including pEGFR, pHER3, PTEN, and FAK. A representative image is shown

As mentioned earlier, the differential gene expression analysis of PDXs used in this study at baseline showed that BCM-3936, BCM-4913, and MC1 (highly responsive to Pan-HER, subgroup 1), differed markedly from the rest of the PDX tumor models (Additional file 2: Figure S1a). Furthermore, IPA analysis showed that genes corresponding to both the PTEN and FAK signaling were in the top canonical pathways. Importantly, a marked reduction of genes related to the PTEN pathway, a very well-described tumor suppressor gene [26, 27], and increased expression of FAK (Additional file 2: Fig. 1b), both representing key components of the AKT/PKB survival pathway [28–30], were observed. In addition to these analyses, Western blots were performed on lysates prepared from PDXs at basal conditions to determine the protein levels and activation status of Pan-HER targets EGFR and HER3, as well as the component of their abovementioned signaling pathway, FAK (Fig. 4). Importantly, PDXs for which the response to Pan-HER was most prominent (i.e., subgroup 1) displayed the highest basal levels of pEGFR, pHER3, and FAK (Fig. 4), indicating a potential dependency on these pathways as a survival asset, in the context of which it is plausible to speculate that the simultaneous inhibition of both EGFR and HER3 may account for the increased efficacy of Pan-HER. Noteworthy, the least responsive PDX tumor model, CVM-4663, displayed the lowest, non-detectable level of EGFR expression/activation.

### Treatment of TNBC PDXs with Pan-HER dramatically reduces activation of EGFR, HER3, and related signaling pathways

**Fig. 5:**
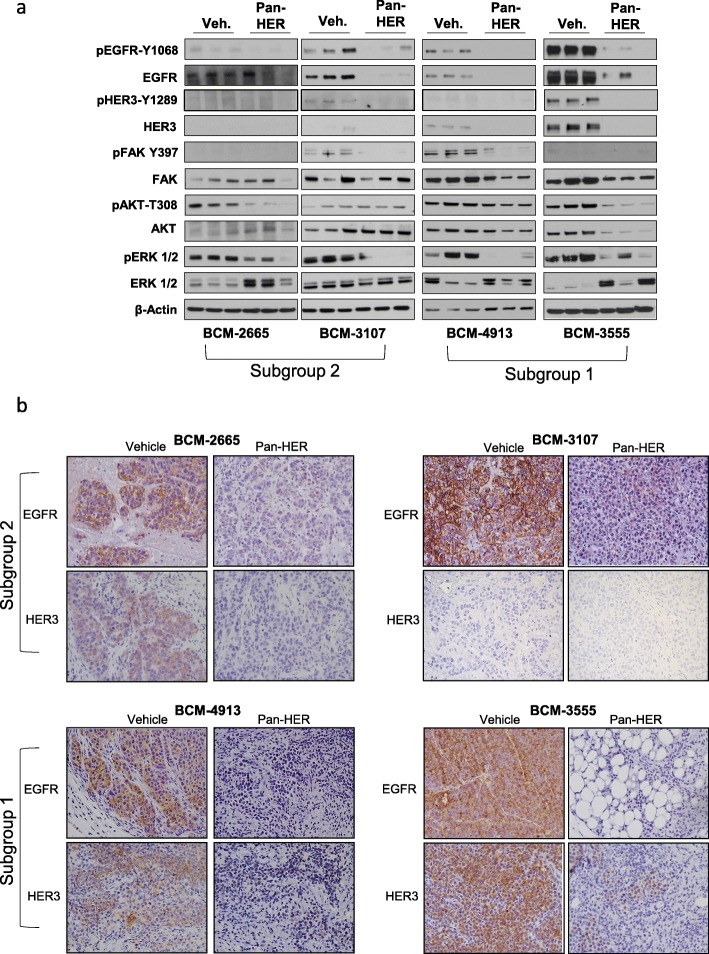
Analysis of HER family members and associated signaling pathways in representative vehicle- and Pan-HER-treated TNBC PDXs. **a** Representative PDX tumor models BCM-2665, BCM-3107 (subgroup 2), BCM-4913, and BMC-3555 (subgroup 1) treated with either vehicle control or Pan-HER for 3 cycles were collected at the end of the experiment (day 21 following the initial injection) and analyzed by Western blot (**a**) or immunohistochemistry (**b**). Western blot analysis of tumor lysates was used to determine HER family members EGFR and HER3, as well as associated signaling pathways including pEGFR, pHER3, FAK, AKT, and ERK pathways. A representative image is shown. β-actin levels were used as loading controls. Each Western blot lane corresponds to an individual PDX/mouse of the designated group. **b** Representative immunohistochemistry analysis of human EGFR and HER3 protein expression was performed in preparations of TNBC tumor samples as described in “Methods”; samples were collected as described above. Counterstain: hematoxylin; here where it says "magnification x 4", it should say "magnification x  20".

To further determine the mechanisms associated with the Pan-HER activity, analysis of PDX samples treated for 3 cycles with either vehicle control or Pan-HER was performed by both Western blot and immunohistochemistry (Fig. 5 and Additional file 2: Figures S2-S16). Exposure to Pan-HER resulted in a marked reduction of target pathway activation in both PDX subgroups. Indeed, Pan-HER induced downmodulation of its targets EGFR and HER3 and their corresponding active phosphorylated forms, pEGFR and pHER3 (Fig. 5 and Additional file 2: Figures S2-S16), in agreement with previously reported results in lung and head and neck cancer cells and cetuximab-resistant non-small cell lung cancer cells [4, 16, 17]. Consistently, inhibition of EGFR and HER3 negatively affected signaling pathway-associated pFAK, pAKT, and pERK (Fig. 5a). Immunohistochemistry (IHC) analysis provided additional evidence of these differences, showing a significant reduction of both EGFR and HER3 in all Pan-HER-treated samples (Fig. 5b and Additional file 2: Figures S2-S16).

**Fig. 6:**
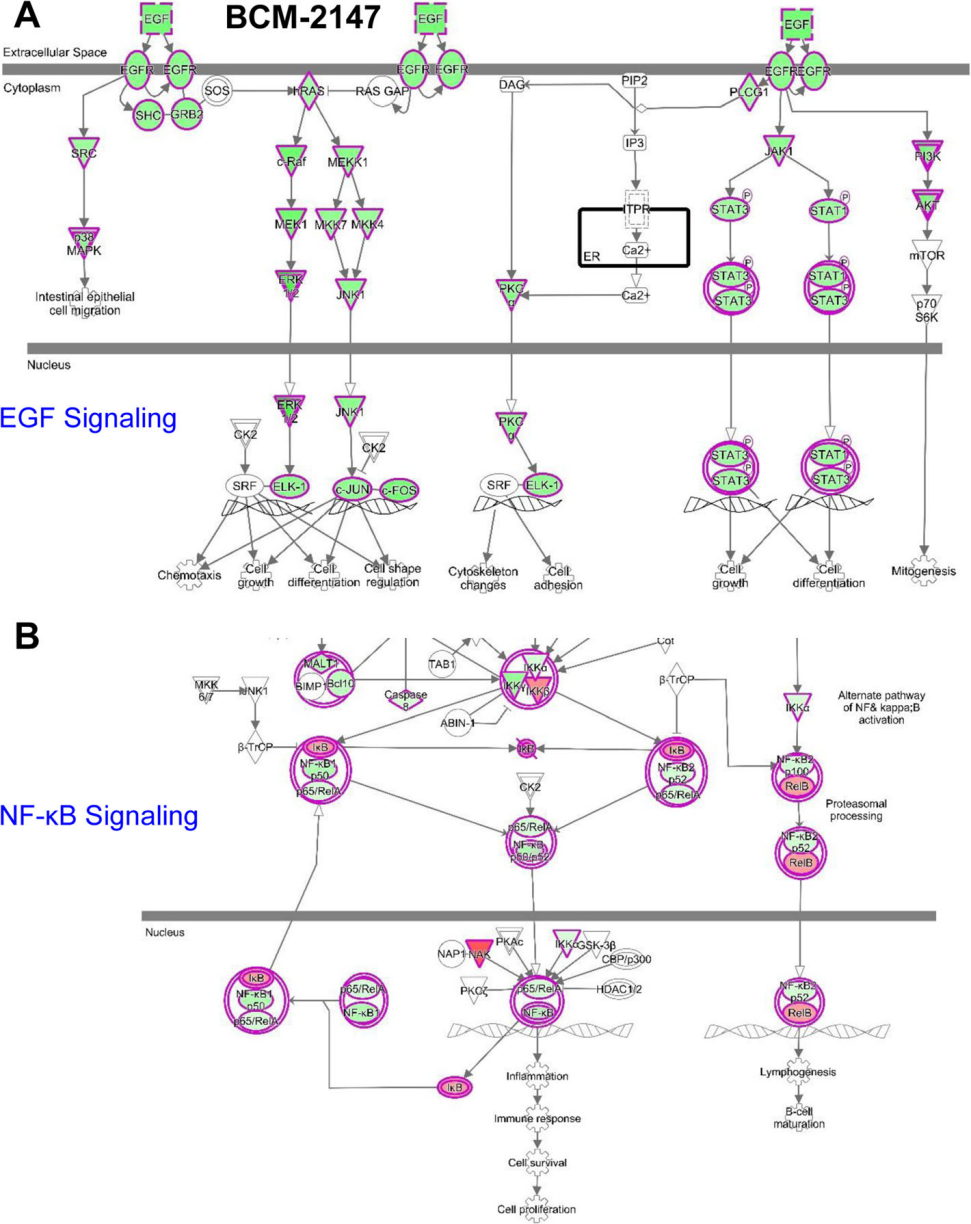
EGFR (**a**) and NF-κB (**b**) pathway-focused RT-PCR gene expression analysis of representative TNBC PDX RNA samples collected before and after Pan-HER treatment. RNA samples corresponding to representative PDX tumor model BCM-2147 (subgroup 2) were extracted from three independent mice (PDX)/group treated with either vehicle control or Pan-HER for 3 cycles at the end of the experiment (day 21 after the initial injection). Triplicate RT-PCR plates were run and relative fold changes of Pan-HER- vs. vehicle control-treated samples for each gene were analyzed by Ingenuity Pathway Analysis (IPA; Qiagen). Genes shown in green represent those significantly downregulated, while those in red upregulated. A twofold change cut-off in gene expression threshold was considered as significantly changed (*P* < 0.001). Further details, as well as a similar analysis performed in three additional PDX models are shown as Supplemental Figures

To further evaluate the impact of Pan-HER-meditated inhibition/downregulation of HER family signaling, a pathway-focused, RT-PCR-based analysis of 88 EGFR-associated genes (Additional file Tables: Supplemental Table S4) followed by pathway analysis by IPA (Qiagen) was performed (Fig. 6 and Additional file 2: Figures S17-S20). Representative PDX models including BCM-3555 and BCM-4913 (subgroup 1), and BCM-2147 and BCM-2665 (subgroup 2) were tested. The RT-PCR analysis was performed using RNA extracted from both vehicle- and Pan-HER-treated tumor tissue collected at the end of the corresponding treatments. Triplicates of each RNA sample, i.e., each PDX model/treatment, were run and analyzed by IPA. As shown in previous figures by IHC and Western blot in all cases, the expression of EGFR was reduced (displayed in green in the corresponding figures). With some differences and/or variations depending the PDX, several genes associated to the EGFR pathway showed also, as expected, significant gene expression downregulation including RAS/cRaf, MEK/ERK, JNK/cJUN/cFOS, and JAK/STAT3. Furthermore, taking into account that (1) EGF triggers NF-κB activation [37–39] and (2) EGFR/NF-κB pathway crosstalk is known to promote resistance to anti-cancer therapies [40–43], a similar pathway-focused analysis was performed to establish the effects of Pan-HER on the NF-κB pathway (Additional file Tables: Supplemental Table S5). As shown in Fig. 6b and Additional file 2: Figures S17-S20, key components of this pathway were affected/downregulated including forms of IKK, IκB, p65/RelA, and others that would have major impact on the capacity of tumor cells to respond to treatment-related stress.

Together, these results demonstrate both the effects of Pan-HER administration on specific HER family targets and pro-survival associated signaling pathways as well as the correlation between pathway activation and therapeutic response, indicating their potential use as surrogate biomarkers for patient selection.

## Discussion

The characteristics of TNBC, i.e., absence of ER/PR/HER2 expression, entail that endocrine and HER2-directed therapies are not appropriate for these types of tumors [44–46]. Furthermore, TNBC will in most cases develop resistance to anthracyclines and taxanes, both considered to be among the most active compounds against breast cancer, thus limiting the therapeutic options even further [47, 48]. With the discovery several years ago of EGFR being overexpressed in TNBCs, a number of new, targeted therapies were developed, including small-molecule tyrosine kinase inhibitors (TKIs) and mAbs. Several of these are now approved and available, including lapatinib, an oral potent HER2 and EGFR inhibitor [49]; afatinib, also a dual HER2/EGFR inhibitor [50, 51]; neratinib, a pan-HER inhibitor that selectively targets HER2, HER4, and EGFR [52, 53], and the recently introduced erlotinib, an EGFR inhibitor [54, 55]. Anti-EGFR mAbs include cetuximab and panitumumab, both of which block ligand-induced phosphorylation of EGFR [56, 57]. However, despite efforts to develop these therapeutic approaches further, these options have not yielded sustained responses and mechanisms of resistance have been encountered after a few months.

In an effort to overcome tumor recurrence and resistance, multiple studies have been addressing the potential use of drug combinations as a way to improve the efficacy of treatment. For example, cetuximab has been clinically tested in combination with cisplatin (metastatic TNBC) [58] and carboplatin (stage IV TNBC) [59] and preclinically tested with ixabepilone and other chemotherapeutic agents [60, 61]. Additional studies have investigated the concept of targeting simultaneously multiple arms of the same HER family, for example by combining a mAb (cetuximab or panitumumab) with small-molecule TKIs [62–64], and established chemotherapeutic agents [60, 61] or other non-competitive mAbs [13].

In the present study, Pan-HER, a mixture of six antibodies simultaneously targeting EGFR, HER2, and HER3 [4, 15], displayed a very promising activity against TNBC xenografts. TNBCs are characterized by the expression of high levels of EGFR [65–67], and the observation that Pan-HER induced a dramatic downregulation of EGFR and HER3, together with their corresponding signaling pathways, is very relevant. In fact, clinical trials with cetuximab combined with either cisplatin [58] or carboplatin [59] showed only moderate improvements vs. chemotherapy alone in terms of overall response rate and progression-free survival, suggesting that targeting EGFR only, even in combination with other therapies, may not be sufficient. Other studies have expanded in the past on the concept of using the blockade of HER family members [13, 68–70]. For example, previous studies in TNBC showed the potential therapeutic advantage of blocking both EGFR and HER3, as a way to improve the efficacy of PI3K-Akt inhibitors [14]. Similarly, the combination of non-competitive anti-EGFR mAbs was found to result in a robust degradation of EGFR also in TNBC cell models, demonstrating a reduction on the tumorigenic growth of cells and derived xenografts [13]. In this context, the present results on the efficacy of Pan-HER on TNBC PDXs showed that the mixture of HER family antibodies was very effective in all the PDX models tested, and more pronouncedly in those tumors that displayed highly active HER family expression and signaling, where no recurrence was observed even after several weeks the treatment ended. On the other hand, the least responsive models corresponded to those showing negative or very low expression of either EGFR or HER3, most notably the former. These observations provide not only a plausible explanation for the response of TNBC tumors to Pan-HER but also an indication of the potential use of EGFR and associated signaling expression as biomarkers for selecting patients who may benefit from targeting these proteins, including cases where Pan-HER may be tested in combination with chemotherapies. In this sense, results from a recent trial (I-SPY 2) evaluating the predictive value of the HER/PI3K/AKT signaling activation/phosphorylation in response to the HER1/2/4 inhibitor neratinib [71], demonstrated that activation of HER family phosphoproteins may be indicative of a response to neratinib.

It is important to note that although not among the most common features observed, relapsed TNBCs have shown some changes in the triple-negative status of the primary tumor type [72]. For example, in a clinical study designed to investigate whether hormonal receptors and HER2 status may be modified throughout tumor progression and therapeutical intervention, it was concluded that these patients experienced changes in the status of hormone receptors and HER2, which could be attributed to adjuvant therapies and may have major impact in survival [72]. Thus, it is plausible to speculate that targeted activation of HER family pathways may help avoid the appearance of resistance. The fact that Pan-HER as a single therapy (inherent combination) was sufficient to achieve complete tumor regression in some tumor models without signs of recurrence makes these findings very promising.

## Conclusions

This preclinical TNBC PDX trial with Pan-HER provides strong justification for further biomarker-guided studies with Pan-HER in TNBC. The finding that tumors were affected rapidly and effectively, with long-lasting results, offers exciting perspectives in the treatment of this aggressive form of breast cancer, for which available treatment options are currently limited.

## Supplementary information

**Additional file 1.** Table 1. Description of the main characteristics of each PDX tumor line are presented including their triple negative (i.e. ER^−^, PR^−^ and HER2^−^) status, and the positive expression of EGFR and HER3 in all of them, with the exception of BCM-4664, which was HER3^−^. Additional information includes, whenever available, TP53 mutations and PAM50 and Pietenpol subtype classification [20, 32–34]. Supplemental Tables 2-3. Sequence analysis of PI3KCA (exons 9 and 20) and EGFR (exons 18, 19, 20 and 21). DNA samples were extracted from all 15 untreated PDX models as described in Additional File Methods. Total DNA was used to perform specific PCR corresponding to PI3KCA (exons 9 and 20) and EGFR (exons 18, 19, 20 and 21). Sequences were analyzed using PI3KCA and EGFR exon sequences from NCBI (EGFR (NM_005228.5) and Multiple Sequence Comparison by Log-Expectation (MUSCLE) program. Supplemental Table 4. Genes included in the pathway-focused RT-PCR analysis corresponding to the human EGFR and NF-kappa B signaling pathways (RealTimePrimers, Elkins Park, PA).

**Additional file 2 **Figure 1. A, Microarray analysis of RNA gene expression corresponding to PDX samples before treatment. Note the differences in expression of PDXs having shown tumor regression (i.e., BCM-3936, BCM-4913 and MC1; Subgroup 1) vs. the rest of the PDXs (Subgroup 2); PDX BCM-4195 does not express EGFR, HER2, or HER3 and was added to the analysis for comparison only. B, gene expression analysis by Ingenuity Pathway Analysis (Qiagen) comparing BCM-3936, BCM-4913 and MC1 (subgroup 1) vs. the remaining PDXs; it shows among the top molecules a marked reduction of genes related to the AKT/PKB survival pathway including the PTEN pathway, and increased expression of PTK2 (FAK). Supplemental Figures 2-16. Time course analysis of the therapeutic response corresponding to each of the 15 TNBC PDXs used in the present study. A, graph displaying the time-course analysis of tumor growth; B, Western blot analysis of HER family members and associated signaling events; and C, IHC of EGFR and HER3 proteins. Low passage TNBC PDX tumor samples (2 mm × 2 mm) were transferred into the right mammary fat pad of mice for engraftment. Once tumors reached an average size of 150-200 mm^3^, mice were randomized (*n* ≥ 3 per group) and treated following the three, one-week cycles design, consisting of 3 times/week IP injection of either formulation buffer (Vehicle control) or Pan-HER (50 mg/kg). Mouse weight was recorded and tumor volumes were measured and calculated as described in Materials & Methods twice weekly. Tumor volume fold change was calculated based on the baseline tumor volumes for each arm. Two-way ANOVA was used for a statistical analysis. At the end of the 3-cycle treatment, the animals were sacrificed and tumors collected for further Western blot and IHC analyses. Supplemental Figures 17-20. EGFR (A) and NF-κB (B) pathway-focused RT-PCR gene expression analysis of representative TNBC PDXs RNA samples collected before and after Pan-HER treatment. RNA samples corresponding to representative PDX tumor model BCM-2147 and BCM-2665 (Subgroup 2), and BCM-3555 and BCM-4913 (Subgroup 1) were extracted from 3 independent mice( PDX)/group treated with either Vehicle control or Pan-HER for 3 cycles at the end of the experiment (day 21 after the initial injection). Triplicate RT-PCR plates were run and relative fold changes of Pan-HER- vs. Vehicle control-treated samples for each gene were analyzed by Ingenuity Pathway Analysis (IPA; Qiagen). Genes shown in green represent those significantly down-regulated, while those in red up-regulated. A 2-fold change cut-off in gene expression threshold was considered as significantly changed (*p* < 0.001). Further details, as well as a similar analysis performed in 3 additional PDX models are shown as Supplemental Figures.

**Additional file 3.** DNA extraction and Sanger sequencing of PIK3CA and EGFR exons

## Data Availability

All the data supporting the results presented in this article are available upon request at the principal investigator’s laboratory.

## Abbreviations

HER: Human epidermal growth factor receptor family
TNBC: Triple-negative breast cancer
PDX: Patient-derived xenografts
ERα: Estrogen receptor alpha
PR: Progesterone receptor
HER2: Epidermal growth factor receptor 2
IACUC: Houston Methodist Research Institute Institutional Animal Care and Use Committee
FAK: Focal adhesion kinase
PTEN: Phosphatase and tensin homolog
TCGA: The Cancer Genome Atlas
TKIs: Tyrosine kinase inhibitors
