# Supplementary material for: Simultaneous targeting of HER family pro-survival signaling with Pan-HER antibody mixture is highly effective in TNBC: a preclinical trial with PDXs
Source: Breast Cancer Res. 2020 May 15;22:48. doi: 10.1186/s13058-020-01280-z (PMC7227035; doi:10.1186/s13058-020-01280-z)
Supplement: Supplementary file 1 — Additional file 1. Table 1. Description of the main characteristics of each PDX tumor line are presented including their triple negative (i.e. ER−, PR− and HER2−) status, and the positive expression of EGFR and HER3 in all of them, with the exception of BCM-4664, which was HER3−. Additional information includes, whenever available, TP53 mutations and PAM50 and Pietenpol subtype classification [20, 32–34]. Supplemental Tables 2-3. Sequence analysis of PI3KCA (exons 9 and 20) and EGFR (exons 18, 19, 20 and 21). DNA samples were extracted from all 15 untreated PDX models as described in Additional File Methods. Total DNA was used to perform specific PCR corresponding to PI3KCA (exons 9 and 20) and EGFR (exons 18, 19, 20 and 21). Sequences were analyzed using PI3KCA and EGFR exon sequences from NCBI (EGFR (NM_005228.5) and Multiple Sequence Comparison by Log-Expectation (MUSCLE) program. Supplemental Table 4. Genes included in the pathway-focused RT-PCR analysis corresponding to the human EGFR and NF-kappa B signaling pathways (RealTimePrimers, Elkins Park, PA). [file 13058_2020_1280_MOESM1_ESM.pdf]

Table S1. Description of the main characteristics of each PDX tumor model used in the present study including their triple negative (i.e. ER-, PR-, and HER2-) status, and the positive expression of EGFR and HER3 in all of them, with the exception of BCM-4664, which was HER3-. Additional information includes, whenever available, TP53 mutations and PAM50 and Pientepol subtype classification.

	PDX lines	ER	PR	EGFR	HER2	HER3	BRCA Mutation	TP53 mutation	k-RAS Mutation	PI3KCA Mutation	cMet Mutation	Drug Resistance (PDX)	Drug Resistance (Clinical)	Lung mets	Pam50	Pietenpol
**1**	BCM-4664	-	-	-/+	-	-		c.747G>T				Dasatinib	Dasatinib Docetaxel	-	Basal	IM
**2**	HCI-002	-	-	+	-	+								-	Basal	nd
**3**	BCM-3887	-	-	+	-	-	+	c.438G>A						+	Basal	M
**4**	BCM-5471	-	-	+	-	+		-						+	Basal	nd
**5**	BCM-2665	-	-	+	-	+		-				Docetaxel	Docetaxel	+	Basal	BL1
**6**	BCM-4013	-	-	+	-	+		c.733G>T						+	Basal	BL2
**7**	BCM-4272	-	-	+	-	+		-				Docetaxel		+	Basal	UNC/BL1
**8**	BCM-3204	-	-	+	-	+		-					Adriamycin	+	Basal	M
**9**	BCM-2147	-	-	+	-	+		-			N375S A1124G		Adriamycin	-	Basal	BL1
**10**	BCM-3107	-	-	+	-	-/+		c.722C>G						-	Basal	M
**11**	HMCC-3186	-	-	+	-	+									nd	nd
**12**	BCM-3936	-	-	+	-	+		c.839G>C						+	Basal	M
**13**	BCM-4913	-	-	+	-	+	+	c.451C>T				Docetaxel	Docetaxel	-	Basal	BL1/nd
**14**	MC1	-	-	+	-	-/+									nd	nd
**15**	HMCC-3555	-	-	+	-	+									nd	nd

nd, not determined; Pietenpol classification: BL1, basal-like 1; BL2 basal-like 2; IM, immunomodulatory; M, Mesenchymal-like; UNC, unclassified; +: EGFR mutation

Table S2: *PIK3CA* Mutation Status. DNA was extracted from tissue samples of all 15 untreated PDX models, and the corresponding exons amplified by PCR by using specific primers described in Supplemental Methods. Amplified fragments were separated in 2% agarose gels, extracted, purified and sequenced. Analysis of the sequences was performed by using PIK3CA NCBI reference sequences NM_006218.3, and Multiple Sequence Comparison by Log-Expectation (MUSCLE) program.

PDX	P539R (exon 9)	E542K (exon 9)	E545K (exon 9)	Y1021C (exon 20)	T1025A (exon 20)	M1043I/V (exon 20)	A1046V (exon 20)	H1047Y/L/R (exon 20)	G1049R (exon 20)
2665-TG4	No	No	No	No	No	No	No	No	No
4664-TG9	No	No	No	No	No	No	No	No	No
3887-TG3	No	No	No	No	No	No	No	No	No
5471-TG8	No	No	No	No	No	No	No	No	No
HCI-002-TG8	No	No	No	No	No	No	No	No	No
4013-TG5	No	No	No	No	No	No	No	No	No
3204-TG4	No	No	No	No	No	No	No	No	No
3107-TG4	No	No	No	No	No	No	No	No	No
4272-TG6	No	No	No	No	No	No	No	No	No
2147-TG5	No	No	No	No	No	No	No	No	No
3936-TG3	No	No	No	No	No	No	No	No	No
MC1-TG6	No	No	No	No	No	No	No	No	No
3186-TG2	No	No	No	No	No	No	No	No	No
4913-TG6	No	No	No	No	No	No	No	No	No
3555-TG2	No	No	No	No	No	No	No	No	No

Table S3: *EGFR* Mutation Status. DNA was extracted from tissue samples of all 15 untreated PDX models, and the corresponding exons amplified by PCR by using specific primers described in Supplemental Methods. Amplified fragments were separated in 2% agarose gels, extracted, purified and sequenced. Analysis of the sequences was performed by using EGFR NCBI reference sequences NM_005228.5, and Multiple Sequence Comparison by Log-Expectation (MUSCLE) program.

PDX	T725T (Exon 18)	E746_A750del (exon 19)	S752_I759del (exon 19)	Inversion of complementary strand (exon 19)	Q787R (exon 20)	T847I (exon 21)	L858R (exon 21)	Q874Q (exon 21) n. 2622 CAG -> CAA
2665-TG4	No	No	No	No	No	No	No	Yes
4664-TG9	No	No	No	No	No	No	No	Yes
3887-TG3	No	No	No	No	No	No	No	Yes
5471-TG8	No	No	No	No	No	No	No	Yes
HCI-002-TG8	No	No	No	No	No	No	No	No
4013-TG5	No	No	No	No	No	No	No	Yes
3204-TG4	No	No	No	No	No	No	No	No
3107-TG4	No	No	No	No	No	No	No	Yes
4272-TG6	No	No	No	No	No	No	No	Yes
2147-TG5	No	No	No	No	No	No	No	Yes
3936-TG3	No	No	No	No	No	No	No	Yes
MC1-TG6	No	No	No	No	No	No	No	Yes
3186-TG2	No	No	No	No	No	No	No	No
4913-TG6	No	No	No	No	No	No	No	Yes
3555-TG2	No	No	No	No	No	No	No	Yes

Table S4. Genes included in the pathway-focused RT-PCR analysis corresponding to the human EGFR and NF-kappa B signaling pathways (RealTimePrimers, Elkins Park, PA).

HEGFR-1 Human EGFR Signaling Primer Library

Well Position	Gene Symbol	Description
A1	AKT1	v-akt murine thymoma viral oncogene homolog 1
A2	AKT2	v-akt murine thymoma viral oncogene homolog 2
A3	AKT3	v-akt murine thymoma viral oncogene homolog 3 (protein kinase B, gamma)
A4	ARAF	v-raf murine sarcoma 3611 viral oncogene homolog
A5	ATF2	activating transcription factor 2
A6	BLC2	B-cell CLL/lymphoma 2
A7	BRAF	v-raf murine sarcoma viral oncogene homolog B1
A8	CAV1	caveolin 1, caveolae protein, 22kDa
A9	CAV2	caveolin 2
A10	CASP3	caspase 3, apoptosis-related cysteine peptidase
A11	CASP9	caspase 9, apoptosis-related cysteine peptidase
A12	CBL	Cbl proto-oncogene, E3 ubiquitin protein ligase
B1	CHUK	conserved helix-loop-helix ubiquitous kinase
B2	CTNNB1	Catenin Beta 1
B3	DUSP1	dual specificity phosphatase 1
B4	DUSP6	dual specificity phosphatase 6
B5	EGF	epidermal growth factor
B6	EGFR	epidermal growth factor receptor
B7	ELK1	ELK1, member of ETS oncogene family
B8	EPS8	epidermal growth factor receptor pathway substrate 8
B9	ERBB2	v-erb-b2 erythroblastic leukemia viral oncogene homolog 2, neuro/glioblastoma derived oncogene homolog
B10	FASLG	Fas ligand (TNF superfamily, member 6)
B11	FOS	FBJ murine osteosarcoma viral oncogene homolog
B12	GAB1	GRB2-associated binding protein 1
C1	GRB2	growth factor receptor-bound protein 2
C2	HRAS	v-Ha-ras Harvey rat sarcoma viral oncogene homolog
C3	IKBKB	inhibitor of kappa light polypeptide gene enhancer in B-cells, kinase beta
C4	IKBKE	inhibitor of kappa light polypeptide gene enhancer in B-cells, kinase epsilon
C5	IKBKG	inhibitor of kappa light polypeptide gene enhancer in B-cells, kinase gamma
C6	JAK1	Janus kinase 1
C7	JAK2	Janus kinase 2
C8	JUN	jun proto-oncogene
C9	KRAS	v-Ki-ras2 Kirsten rat sarcoma viral oncogene homolog
C10	MAP2	microtubule-associated protein 2

C11	MAP2K1	mitogen-activated protein kinase kinase 1
C12	MAP2K2	mitogen-activated protein kinase kinase 2
D1	MAP2K3	mitogen-activated protein kinase kinase 3
D2	MAP2K4	mitogen-activated protein kinase kinase 4
D3	MAP2K5	mitogen-activated protein kinase kinase 5
D4	MAP2K6	mitogen-activated protein kinase kinase 6
D5	MAP2K7	mitogen-activated protein kinase kinase 7
D6	MAP3K1	mitogen-activated protein kinase kinase kinase 1, E3 ubiquitin protein ligase
D7	MAP3K2	mitogen-activated protein kinase kinase kinase 2
D8	MAP3K3	mitogen-activated protein kinase kinase kinase 3
D9	MAPK1	mitogen-activated protein kinase 1
D10	MAPK10	mitogen-activated protein kinase 10
D11	MAPK12	mitogen-activated protein kinase 12
D12	MAPK14	mitogen-activated protein kinase 14
E1	MAPK3	mitogen-activated protein kinase 3
E2	MAPK8	mitogen-activated protein kinase 8
E3	MAPK9	mitogen-activated protein kinase 9
E4	MBP	myelin basic protein
E5	MRAS	muscle RAS oncogene homolog
E6	MYC	V-myc myelocytomatosis viral oncogene homolog (avian)
E7	NFKB1	nuclear factor of kappa light polypeptide gene enhancer in B-cells 1
E8	NRAS	neuroblastoma RAS viral (v-ras) oncogene homolog
E9	PAK1	p21 protein (Cdc42/Rac)-activated kinase 1
E10	PDGFB	platelet-derived growth factor beta polypeptide
E11	PIKCA	phosphoinositide-3-kinase, catalytic, alpha polypeptide
E12	PIK3R1	phosphoinositide-3-kinase, regulatory subunit 1 (alpha)
F1	PIK3R2	phosphoinositide-3-kinase, regulatory subunit 2 (beta)
F2	PIK3R3	phospholipase C, beta 3 (phosphatidylinositol- specific)
F3	PLCG1	phospholipase C, gamma 1
F4	PRKCA	protein kinase C, alpha
F5	PRKCB	protein kinase C, beta
F6	PRKCD	protein kinase C, delta
F7	PRKCE	protein kinase C, epsilon
F8	PRKCG	protein kinase C, gamma
F9	PTEN	phosphatase and tensin homolog
F10	RAC1	ras-related C3 botulinum toxin substrate 1 (rho family, small GTP binding protein Rac1)
F11	RAC2	ras-related C3 botulinum toxin substrate 2 (rho family, small GTP binding protein Rac2)
F12	RAC3	ras-related C3 botulinum toxin substrate 3 (rho family, small GTP binding protein Rac3)
G1	RAF1	v-raf-1 murine leukemia viral oncogene homolog 1
G2	RAP1A	RAP1A, member of RAS oncogene family

G3	REL	v-rel reticuloendotheliosis viral oncogene homolog (avian)
G4	RELA	v-rel reticuloendotheliosis viral oncogene homolog A (avian)
G5	RELB	v-rel reticuloendotheliosis viral oncogene homolog B
G6	RHOA	ras homolog family member A
G7	RHOB	ras homolog family member B
G8	RHOC	ras homolog family member C
G9	RHOD	ras homolog family member D
G10	RHOG	ras homolog family member G
G11	RRAS	related RAS viral (r-ras) oncogene homolog
G12	SHC1	SHC (Src homology 2 domain containing) transforming protein 1
H1	SIPA1	signal-induced proliferation-associated 1
H2	SRC	v-src sarcoma (Schmidt-Ruppin A-2) viral oncogene homolog (avian)
H3	STAT1	signal transducer and activator of transcription 1, 91kDa
H4	STAT3	signal transducer and activator of transcription 3 (acute-phase response factor)
H5	ACTB	Actin, beta
H6	B2M	Beta-2-microglobulin
H7	GAPDH	Glyceraldehyde-3-phosphate dehydrogenase
H8	GUSB	Glucuronidase, beta
H9	HPRT1	Hypoxanthine phosphoribosyltransferase 1
H10	PGK1	Phosphoglycerate kinase 1
H11	PPIA	Peptidylprolyl isomerase A
H12	RPL13A	Ribosomal protein L13a

Human NFKB Signaling Primer Library

Well Position	Gene Symbol	Description
A1	AGT	Angiotensinogen
A2	AKT1	V-akt murine thymoma viral oncogene homolog 1
A3	ATF1	Activating transcription factor 1
A4	BCL10	B-cell CLL/lymphoma 10
A5	BCL2	B-cell CLL/lymphoma 2
A6	BCL2L1	BCL2-like 1
A7	BCL3	B-cell CLL/lymphoma 3
A8	BIRC2	Baculoviral IAP repeat-containing 2
A9	BIRC4	Baculoviral IAP repeat-containing 4
A10	CASP1	Caspase 1, apoptosis-related cysteine peptidase
A11	CASP8	Caspase 8, apoptosis-related cysteine peptidase
A12	CCL2	Chemokine (C-C motif) ligand 2
B1	CD40	CD40 molecule, TNF receptor superfamily member 5
B2	CFLAR	CASP8 and FADD-like apoptosis regulator
B3	CHUK	Conserved helix-loop-helix ubiquitous kinase
B4	CSF1	Colony stimulating factor 1 (macrophage)
B5	CSF2	Colony stimulating factor 2 (granulocyte- macrophage)
B6	CSF3	Colony stimulating factor 3 (granulocyte)
B7	EDARADD	EDAR-associated death domain
B8	EDG2	Lysophosphatidic acid receptor 1
B9	EGR1	Early growth response 1
B10	ELK1	ELK1, member of ETS oncogene family
B11	F2R	Coagulation factor II (thrombin) receptor
B12	FADD	Fas (TNFRSF6)-associated via death domain
C1	FASLG	Fas ligand (TNF superfamily, member 6)
C2	FOS	V-fos FBJ murine osteosarcoma viral oncogene
C3	GJA1	Gap junction protein, alpha 1, 43kDa
C4	HMOX1	Heme oxygenase (decycling) 1
C5	HTR2B	5-hydroxytryptamine (serotonin) receptor 2B
C6	ICAM1	Intercellular adhesion molecule 1 (CD54)
C7	IFNA1	Interferon, alpha 1
C8	IFNAB1	Interferon, beta 1
C9	IFNG	Interferon, gamma
C10	IKBKB	Inhibitor of kappa light polypeptide gene enhancer in B-cells, kinase beta
C11	IKBKE	Inhibitor of kappa light polypeptide gene enhancer in B-cells, kinase epsilon
C12	IKBKG	Inhibitor of kappa light polypeptide gene enhancer in B-cells, kinase gamma
D1	IL10	Interleukin 10

D2	IL12A	Interleukin 12A
D3	IL12B	Interleukin 12B
D4	IL1A	Interleukin 1, alpha
D5	IL1B	Interleukin 1, beta
D6	IL1R1	Interleukin 1 receptor, type I
D7	IL6	Interleukin 6 (interferon, beta 2)
D8	IL8	Interleukin 8
D9	IRAK1	Interleukin-1 receptor-associated kinase 1
D10	IRAK2	Interleukin-1 receptor-associated kinase 2
D11	JUN	Jun oncogene
D12	LTA	Lymphotoxin alpha (TNF superfamily, member 1)
E1	LTB	Lymphotoxin beta (TNF superfamily, member 3)
E2	LTBR	Lymphotoxin beta receptor
E3	MALT1	Mucosa associated lymphoid tissue lymphoma translocation gene 1
E4	MAP3K1	Mitogen-activated protein kinase kinase kinase 1
E5	MMP7	Matrix metallopeptidase 7 (matrilysin, uterine)
E6	MMP9	Matrix metallopeptidase 9
E7	NFKB1	Nuclear factor of kappa light polypeptide gene enhancer in B-cells 1 (p105)
E8	NFKB2	Nuclear factor of kappa light polypeptide gene enhancer in B-cells 2 (p49/p100)
E9	NFKBIA	Nuclear factor of kappa light polypeptide gene enhancer in B-cells inhibitor, alpha
E10	NLRP12	NLR family, pyrin domain containing 12
E11	NOD1	Nucleotide-binding oligomerization domain containing 1
E12	PPM1A	Protein phosphatase 1A (formerly 2C), alpha isoform
F1	RAF1	V-raf-1 murine leukemia viral oncogene homolog 1
F2	REL	V-rel reticuloendotheliosis viral oncogene homolog (avian)
F3	RELA	V-rel reticuloendotheliosis viral oncogene homolog A
F4	RELB	V-rel reticuloendotheliosis viral oncogene homolog B
F5	RHOA	Ras homolog gene family, member A
F6	RIPK1	Receptor (TNFRSF)-interacting serine-threonine kinase 1
F7	SELL	Selectin L
F8	SELP	Selectin P
F9	STAT1	Signal transducer and activator of transcription 1
F10	TBK1	TANK binding kinase 1
F11	TICAM1	Toll-like receptor adaptor molecule 1
F12	TICAM2	Transmembrane emp24 protein transport domain containing 7
G1	TLR1	Toll-like receptor 1
G2	TLR2	Toll-like receptor 2
G3	TLR3	Toll-like receptor 3
G4	TLR4	Toll-like receptor 4
G5	TLR7	Toll-like receptor 7
G6	TLR8	Toll-like receptor 8

G7	TLR9	Toll-like receptor 9
G8	TMED4	Transmembrane emp24 protein transport domain containing 4
G9	TNF	Tumor necrosis factor (TNF superfamily, member 2)
G10	TNFAIP3	Tumor necrosis factor, alpha-induced protein 3
G11	TNFRSF10A	Tumor necrosis factor receptor superfamily, member 10a
G12	TNFRSF10B	Tumor necrosis factor receptor superfamily, member 10b
H1	TNFRSF1A	Tumor necrosis factor receptor superfamily, member 1A
H2	TNFSF10	Tumor necrosis factor (ligand) superfamily, member 10
H3	TNFSF15	Tumor necrosis factor (ligand) superfamily, member 15
H4	TRADD	TNFRSF1A-associated via death domain
H5	ACTB	Actin, beta
H6	B2M	Beta-2-microglobulin
H7	GAPD	Glyceraldehyde phosphate dehydrogenase
H8	GUSB	Glucuronidase, beta
H9	HPRT1	Hypoxanthine phosphoribosyltransferase 1
H10	PGK1	Phosphoglycerate kinase 1
H11	PPIA	Peptidylprolyl isomerase A
H12	RPL13A	Ribosomal protein L13a
