# Supplementary material for: Simultaneous targeting of HER family pro-survival signaling with Pan-HER antibody mixture is highly effective in TNBC: a preclinical trial with PDXs
Source: Breast Cancer Res. 2020 May 15;22:48. doi: 10.1186/s13058-020-01280-z (PMC7227035; doi:10.1186/s13058-020-01280-z)

Figure S1

**a**

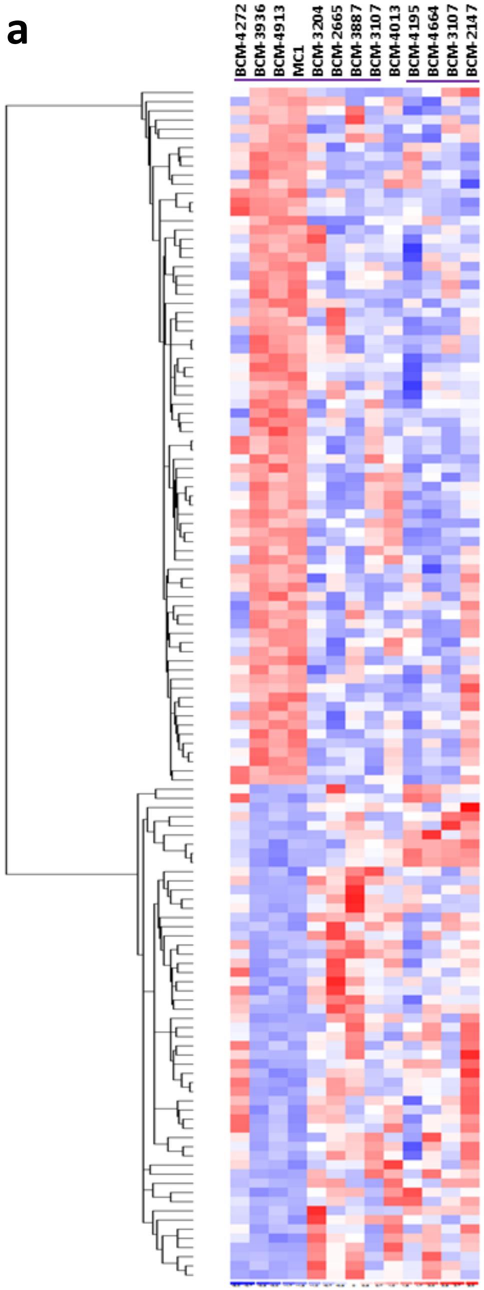


**b**

**Top Canonical Pathways**

| Name | p-value | Overlap | |
|---|---|---|---|
| PTEN Signaling | 3.37E-03 | 3.4 % | 4/117 |
| Epithelial Adherens Junction Signaling | 6.51E-03 | 2.8 % | 4/141 |
| Neuregulin Signaling | 1.02E-02 | 3.5 % | 3/85 |
| Formaldehyde Oxidation II (Glutathione-dependent) | 1.05E-02 | 50.0 % | 1/2 |
| FAK Signaling | 1.05E-02 | 3.5 % | 3/86 |

**Top Molecules**

**Fold Change up-regulated**

| Molecules | Exp. Value |
|---|---|
| PYGL | ↑ 3.300 |
| GALNT7* | ↑ 3.070 |
| NRAS | ↑ 2.940 |
| SVIL | ↑ 2.590 |
| CMTM3 | ↑ 2.530 |
| METTL21A | ↑ 2.450 |
| ID2 | ↑ 2.440 |
| PTK2 | ↑ 2.380 |
| LGMN | ↑ 2.230 |
| RNF144B | ↑ 2.180 |

**Fold Change down-regulated**

| Molecules | Exp. Value |
|---|---|
| HOXB13 | ↓ -27.580 |
| PTEN* | ↓ -20.330 |
| PARP11 | ↓ -16.470 |
| PAG1 | ↓ -10.560 |
| TOX | ↓ -10.170 |
| NRGN | ↓ -9.320 |
| MAP9 | ↓ -6.290 |
| FXYD6 | ↓ -5.820 |
| SLC7A11* | ↓ -5.760 |
| TRIQK | ↓ -5.680 |

# Figure S2

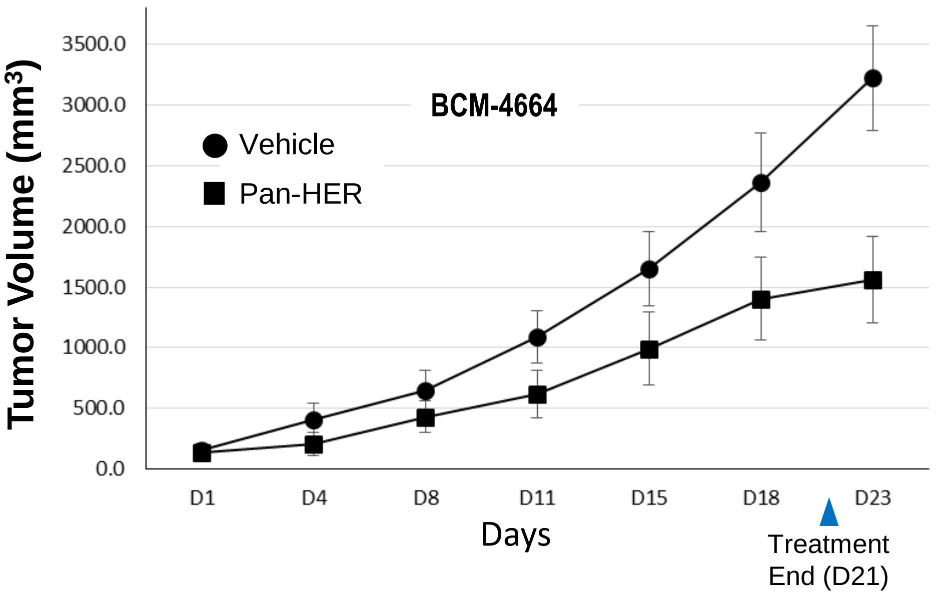
BCM-4664
Vehicle
Pan-HER
Tumor Volume (mm³)
3500.0
3000.0
2500.0
2000.0
1500.0
1000.0
500.0
0.0
D1
D4
D8
D11
D15
D18
D23
Days
Treatment
End (D21)

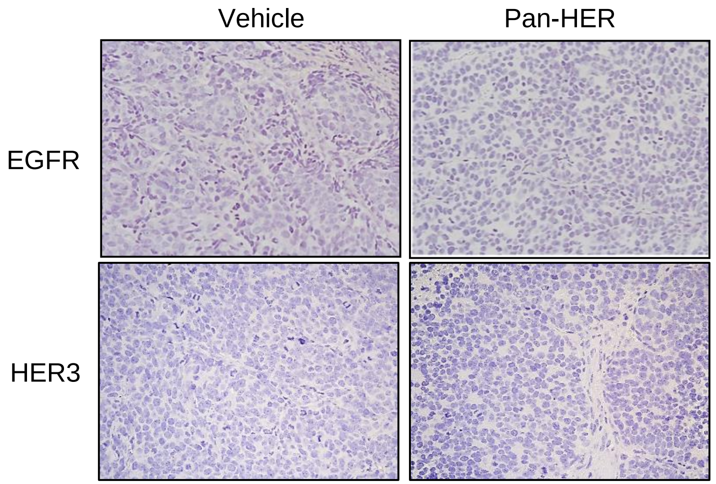
Vehicle
Pan-HER
EGFR
HER3

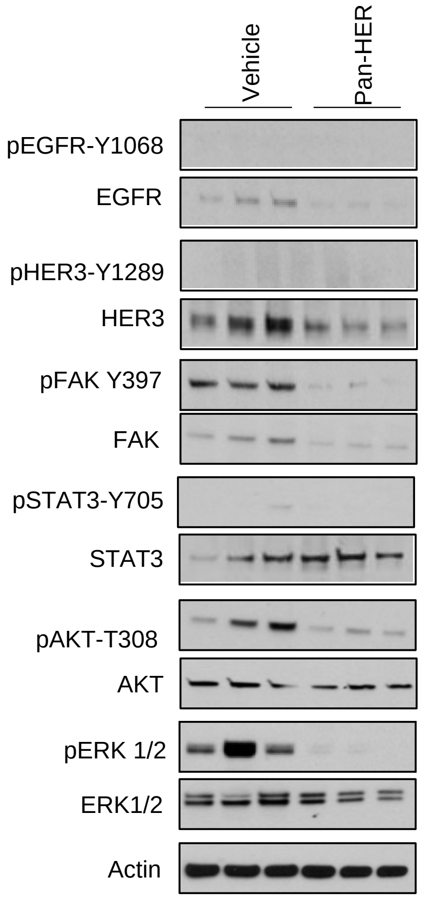
Vehicle
Pan-HER
pEGFR-Y1068
EGFR
pHER3-Y1289
HER3
pFAK Y397
FAK
pSTAT3-Y705
STAT3
pAKT-T308
AKT
pERK 1/2
ERK1/2
Actin

Figure S3

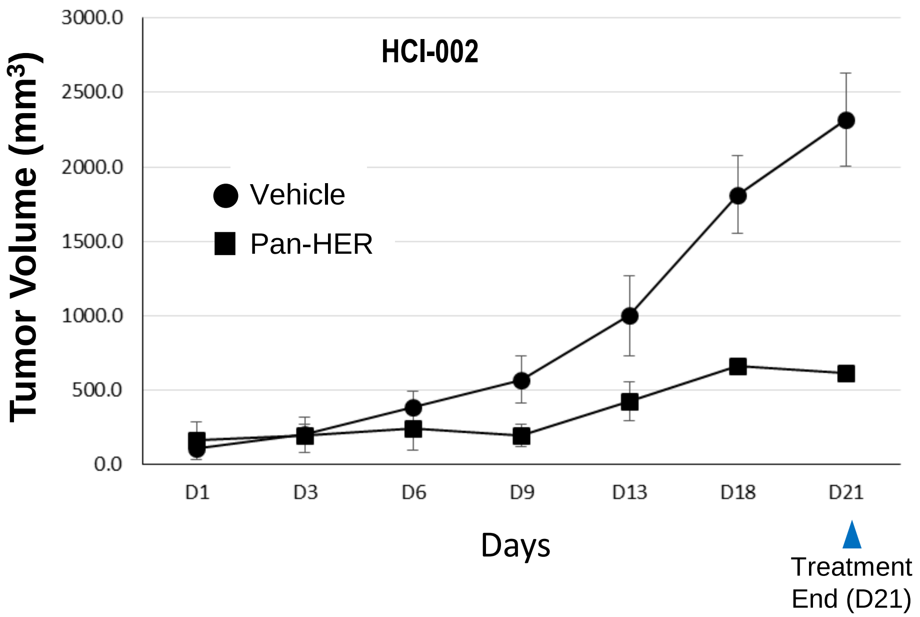
HCI-002
Tumor Volume (mm³)
3000.0
2500.0
2000.0
1500.0
1000.0
500.0
0.0
Vehicle
Pan-HER
D1
D3
D6
D9
D13
D18
D21
Days
Treatment
End (D21)

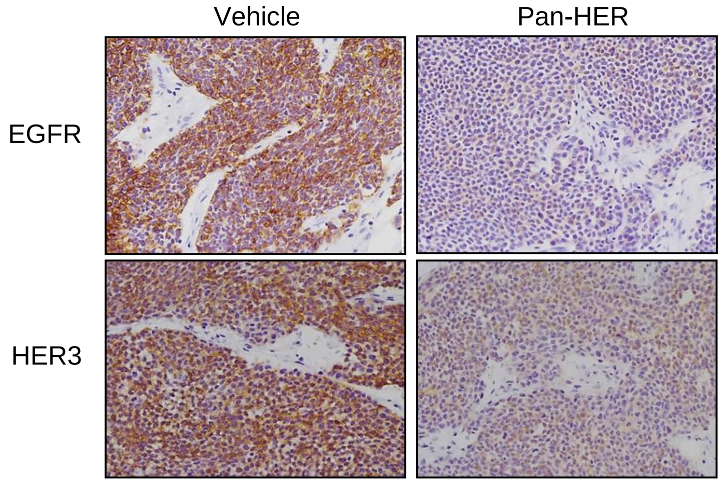
Vehicle
Pan-HER
EGFR
HER3

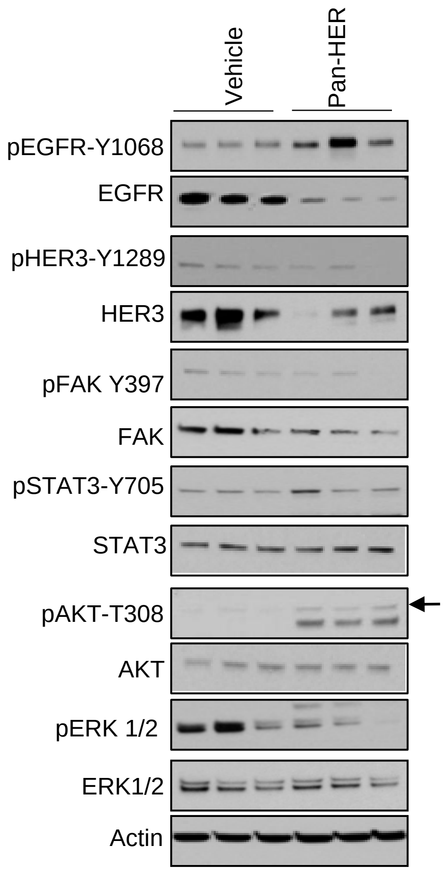
Vehicle
Pan-HER
pEGFR-Y1068
EGFR
pHER3-Y1289
HER3
pFAK Y397
FAK
pSTAT3-Y705
STAT3
pAKT-T308
AKT
pERK 1/2
ERK1/2
Actin

Figure S4

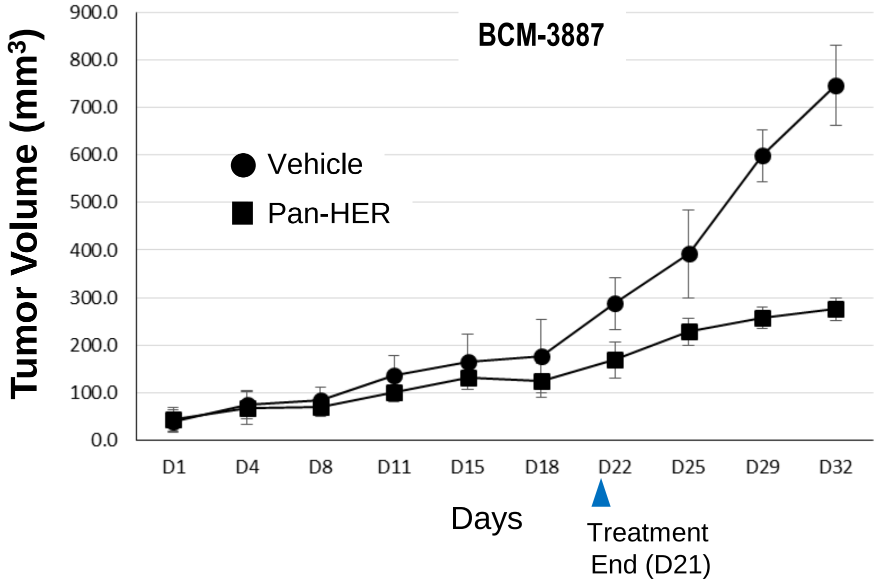
BCM-3887
Tumor Volume (mm3)
900.0
800.0
700.0
600.0
500.0
400.0
300.0
200.0
100.0
0.0
Vehicle
Pan-HER
D1
D4
D8
D11
D15
D18
D22
D25
D29
D32
Days
Treatment End (D21)

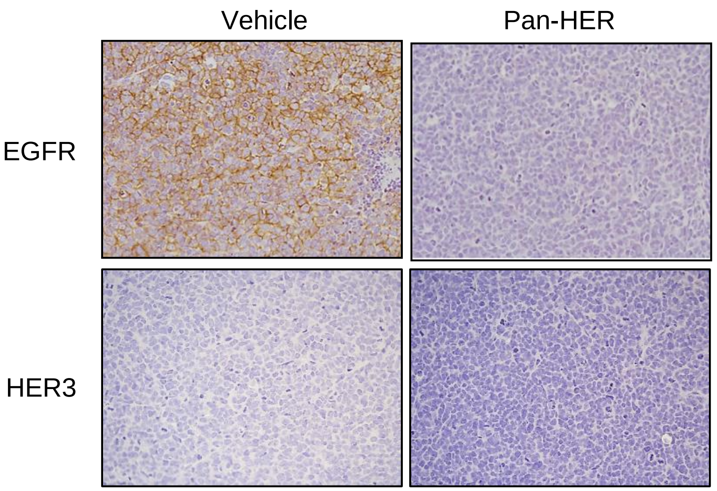
Vehicle
Pan-HER
EGFR
HER3

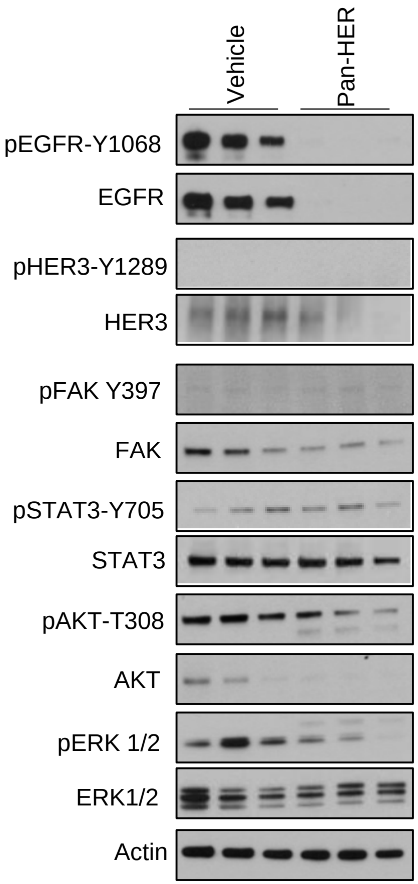
Vehicle
Pan-HER
pEGFR-Y1068
EGFR
pHER3-Y1289
HER3
pFAK Y397
FAK
pSTAT3-Y705
STAT3
pAKT-T308
AKT
pERK 1/2
ERK1/2
Actin

# Figure S5

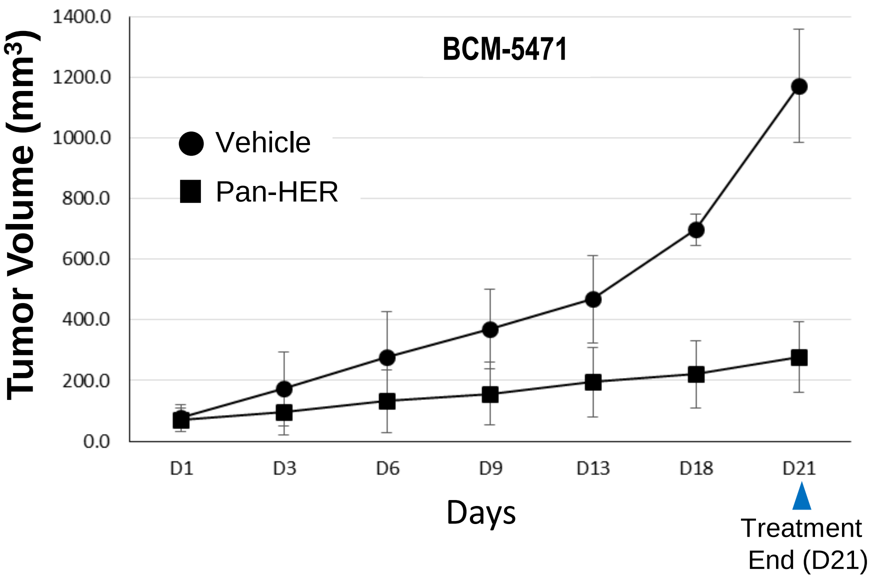
BCM-5471
Tumor Volume (mm3)
1400.0
1200.0
1000.0
800.0
600.0
400.0
200.0
0.0
Vehicle
Pan-HER
D1
D3
D6
D9
D13
D18
D21
Days
Treatment End (D21)

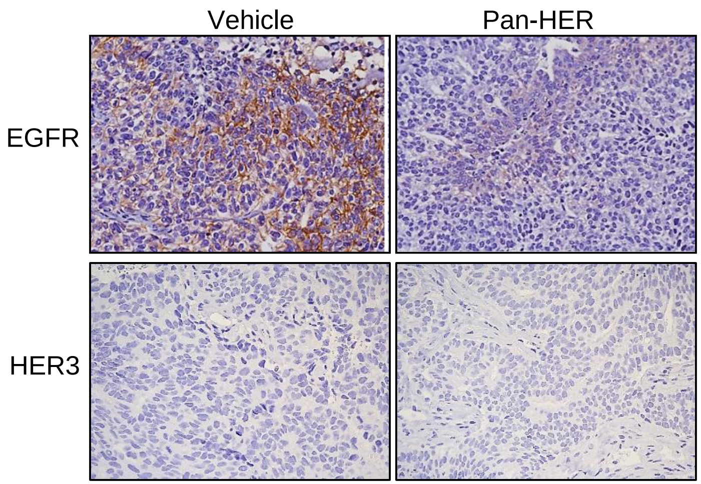
Vehicle
Pan-HER
EGFR
HER3

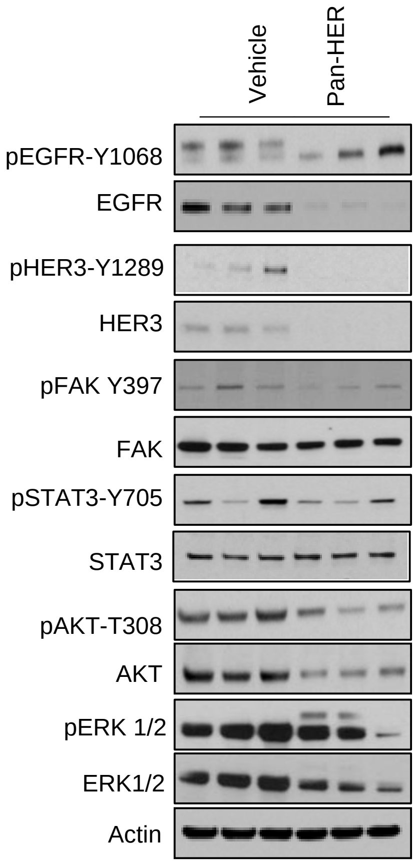
Vehicle
Pan-HER
pEGFR-Y1068
EGFR
pHER3-Y1289
HER3
pFAK Y397
FAK
pSTAT3-Y705
STAT3
pAKT-T308
AKT
pERK 1/2
ERK1/2
Actin

Figure S6

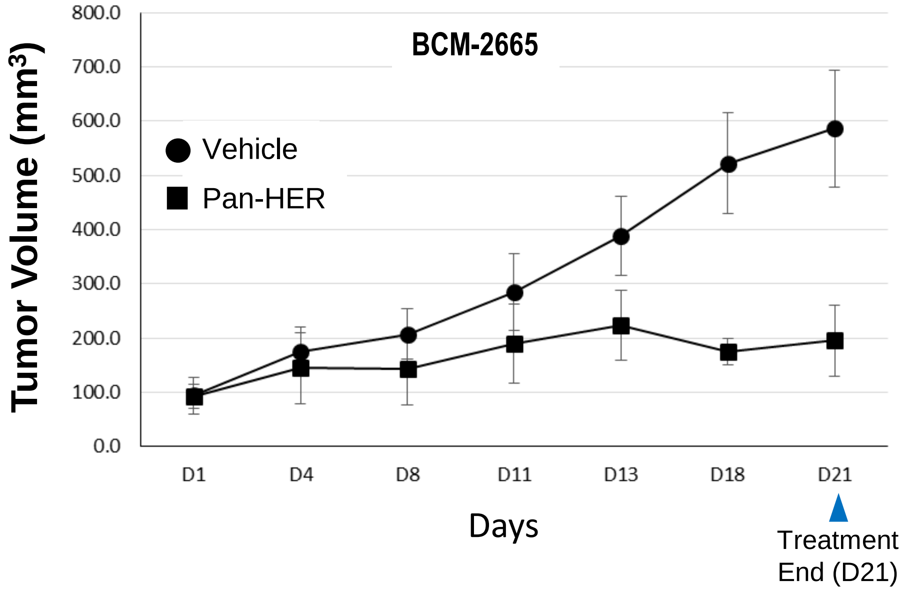
BCM-2665
Tumor Volume (mm³)
800.0
700.0
600.0
500.0
400.0
300.0
200.0
100.0
0.0
Vehicle
Pan-HER
D1
D4
D8
D11
D13
D18
D21
Days
Treatment End (D21)

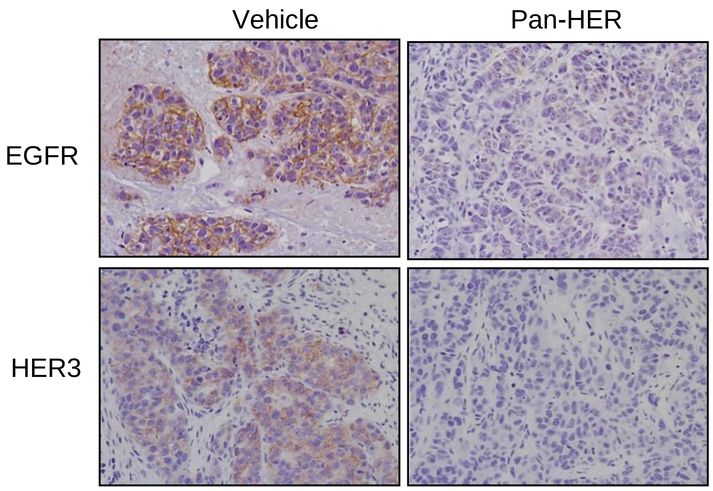
Vehicle
Pan-HER
EGFR
HER3

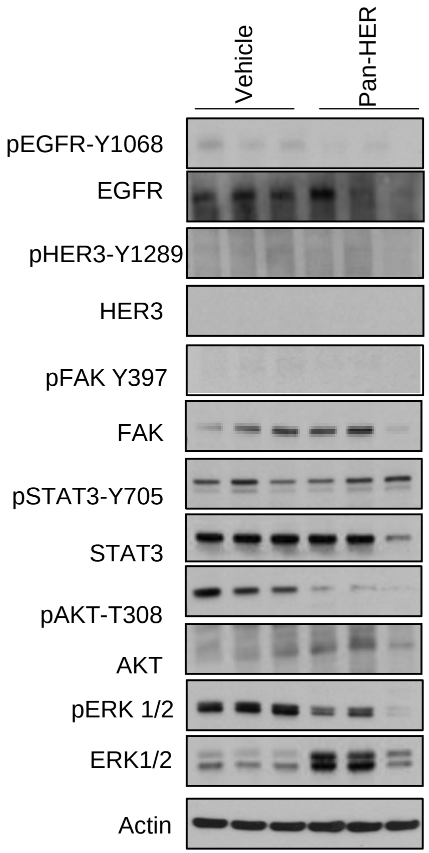
Vehicle
Pan-HER
pEGFR-Y1068
EGFR
pHER3-Y1289
HER3
pFAK Y397
FAK
pSTAT3-Y705
STAT3
pAKT-T308
AKT
pERK 1/2
ERK1/2
Actin

Figure S7

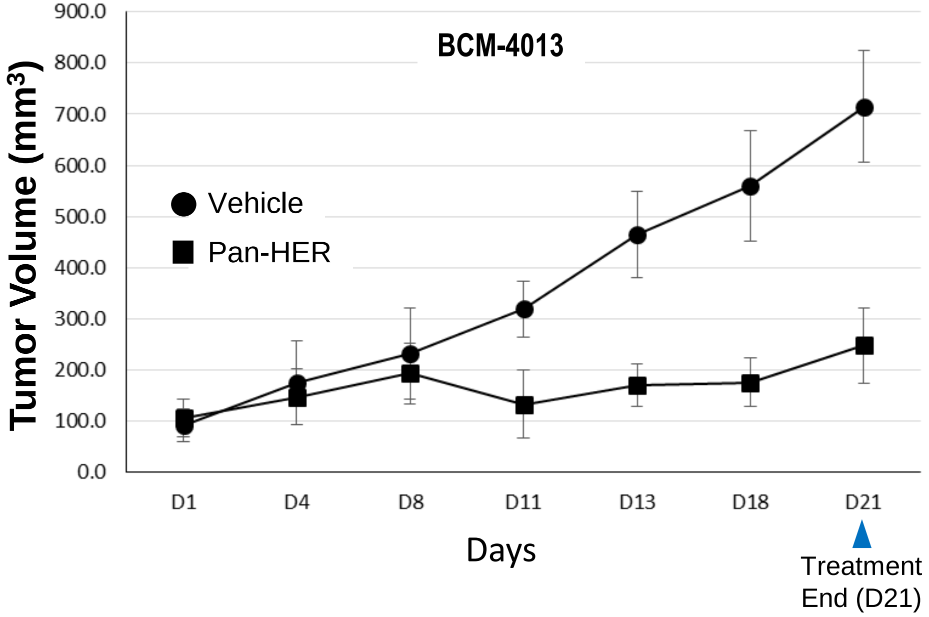

BCM-4013
Tumor Volume (mm3)
900.0
800.0
700.0
600.0
500.0
400.0
300.0
200.0
100.0
0.0
Vehicle
Pan-HER
D1
D4
D8
D11
D13
D18
D21
Days
Treatment End (D21)


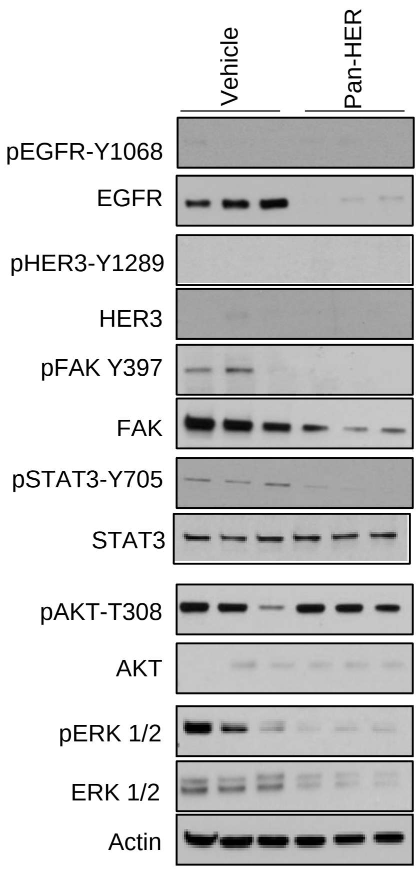

Vehicle
Pan-HER
pEGFR-Y1068
EGFR
pHER3-Y1289
HER3
pFAK Y397
FAK
pSTAT3-Y705
STAT3
pAKT-T308
AKT
pERK 1/2
ERK 1/2
Actin


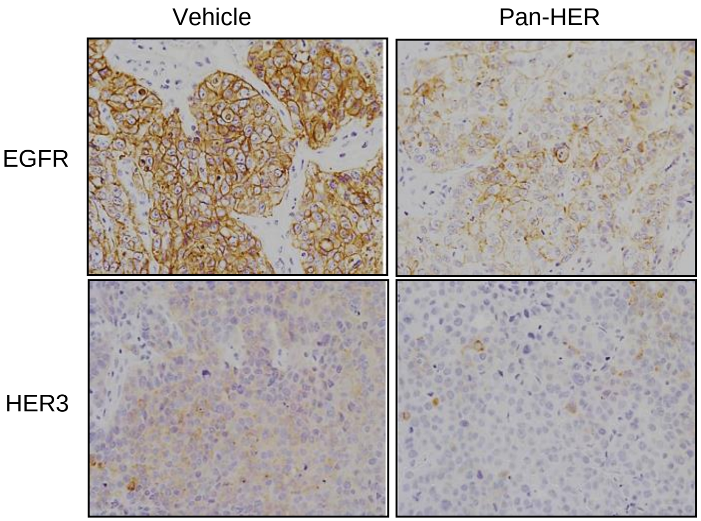

Vehicle
Pan-HER
EGFR
HER3

Figure S8

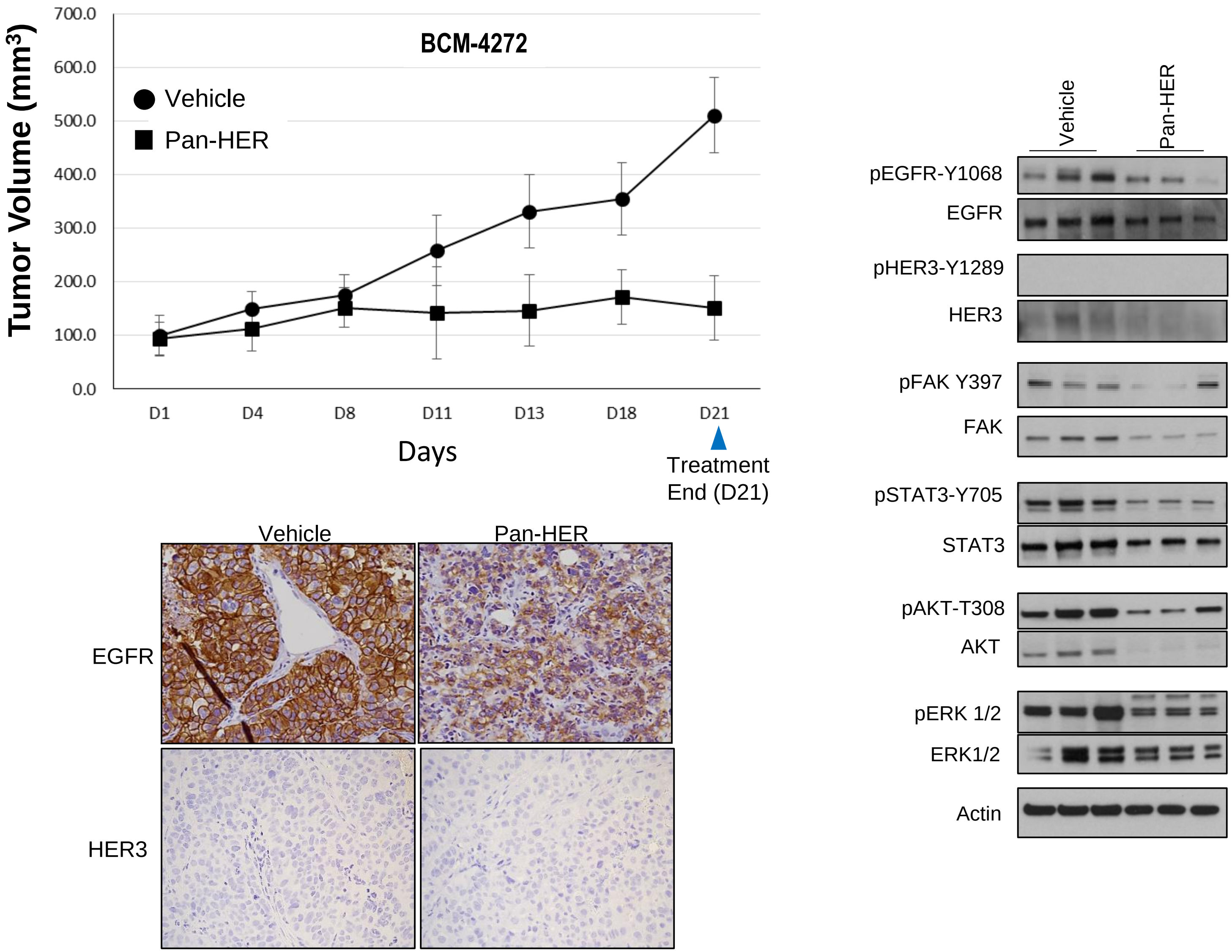

BCM-4272
Tumor Volume (mm³)
700.0
600.0
500.0
400.0
300.0
200.0
100.0
0.0
Vehicle
Pan-HER
D1
D4
D8
D11
D13
D18
D21
Days
Treatment End (D21)
Vehicle
Pan-HER
EGFR
HER3
pEGFR-Y1068
EGFR
pHER3-Y1289
HER3
pFAK Y397
FAK
pSTAT3-Y705
STAT3
pAKT-T308
AKT
pERK 1/2
ERK1/2
Actin

# Figure S9

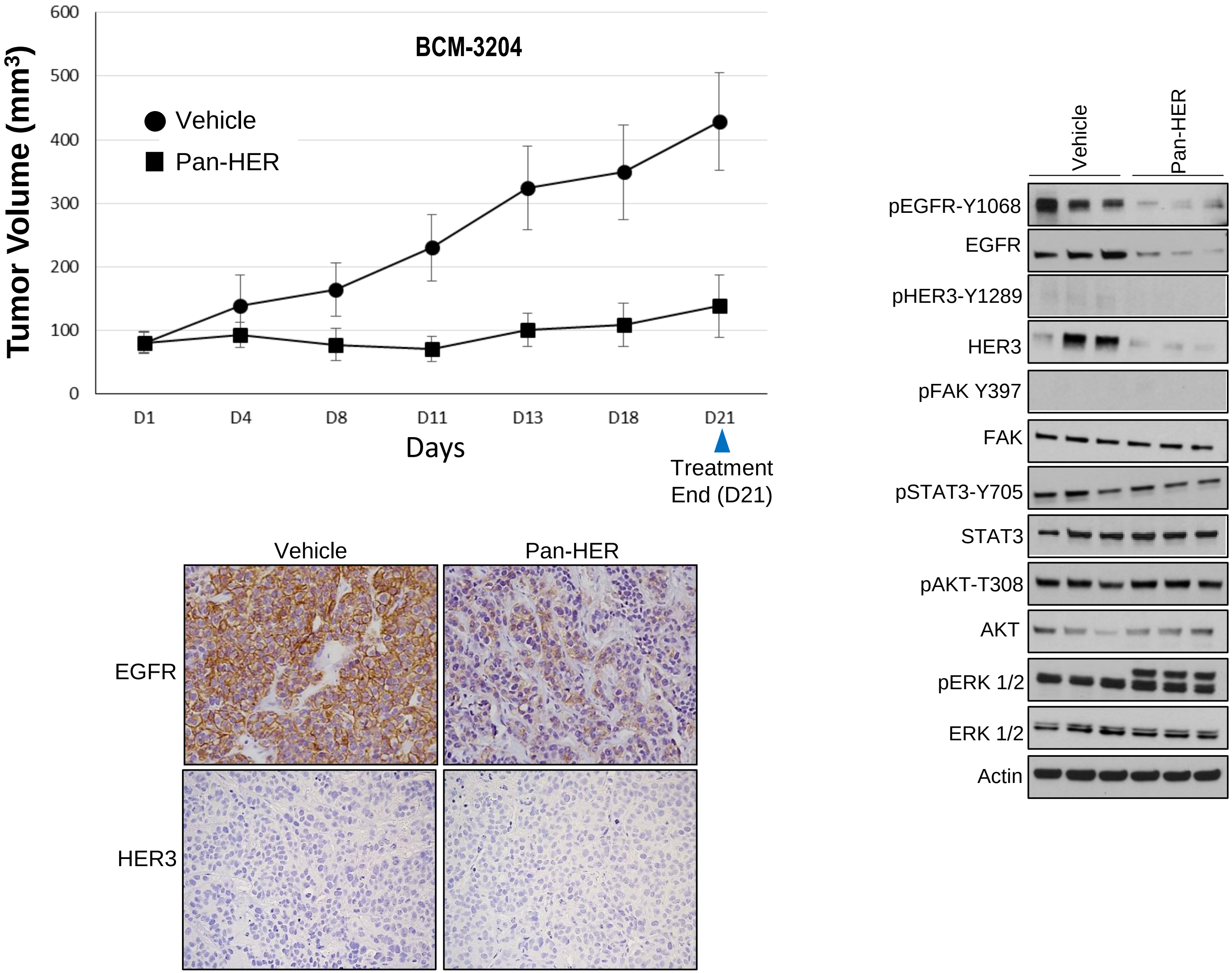

BCM-3204
Tumor Volume (mm³)
600
500
400
300
200
100
0
Vehicle
Pan-HER
D1
D4
D8
D11
D13
D18
D21
Days
Treatment
End (D21)
Vehicle
Pan-HER
EGFR
HER3
Vehicle
Pan-HER
pEGFR-Y1068
EGFR
pHER3-Y1289
HER3
pFAK Y397
FAK
pSTAT3-Y705
STAT3
pAKT-T308
AKT
pERK 1/2
ERK 1/2
Actin

# Figure S10

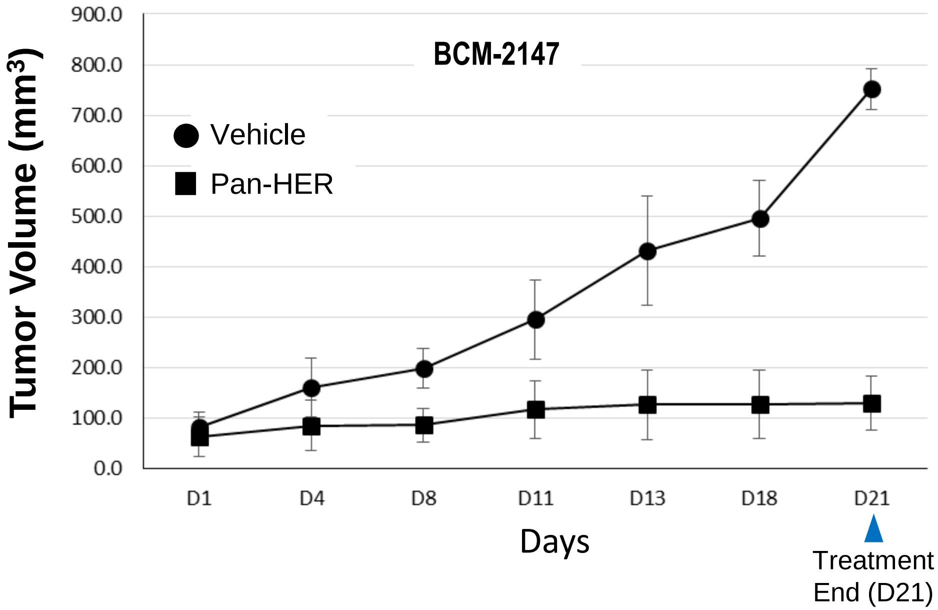

BCM-2147
Tumor Volume (mm3)
900.0
800.0
700.0
600.0
500.0
400.0
300.0
200.0
100.0
0.0
Vehicle
Pan-HER
D1
D4
D8
D11
D13
D18
D21
Days
Treatment
End (D21)


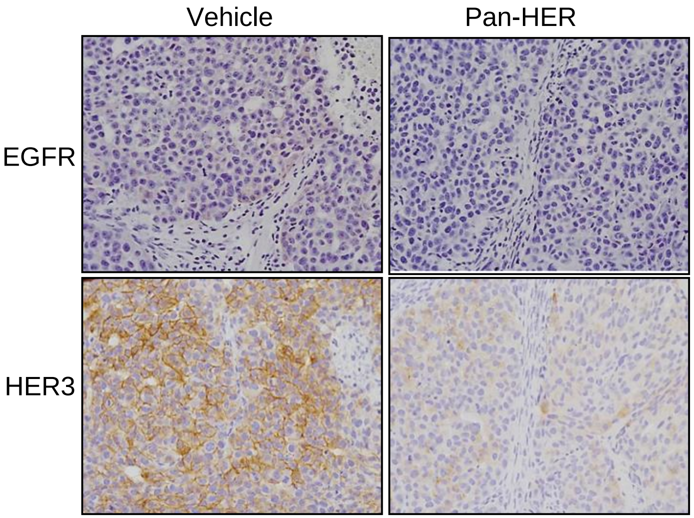

Vehicle
Pan-HER
EGFR
HER3


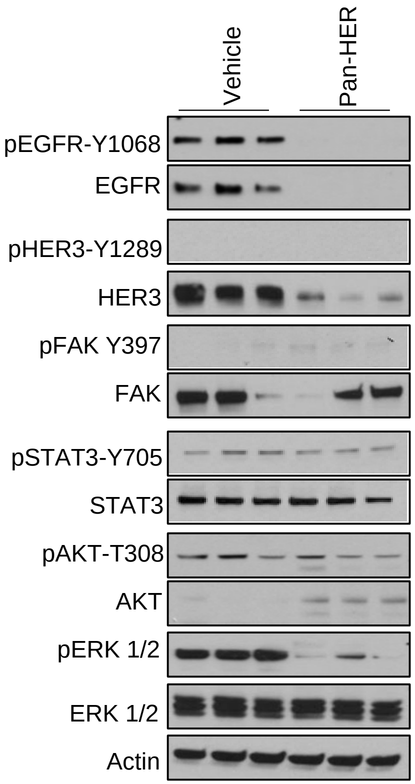

Vehicle
Pan-HER
pEGFR-Y1068
EGFR
pHER3-Y1289
HER3
pFAK Y397
FAK
pSTAT3-Y705
STAT3
pAKT-T308
AKT
pERK 1/2
ERK 1/2
Actin

# Figure S11

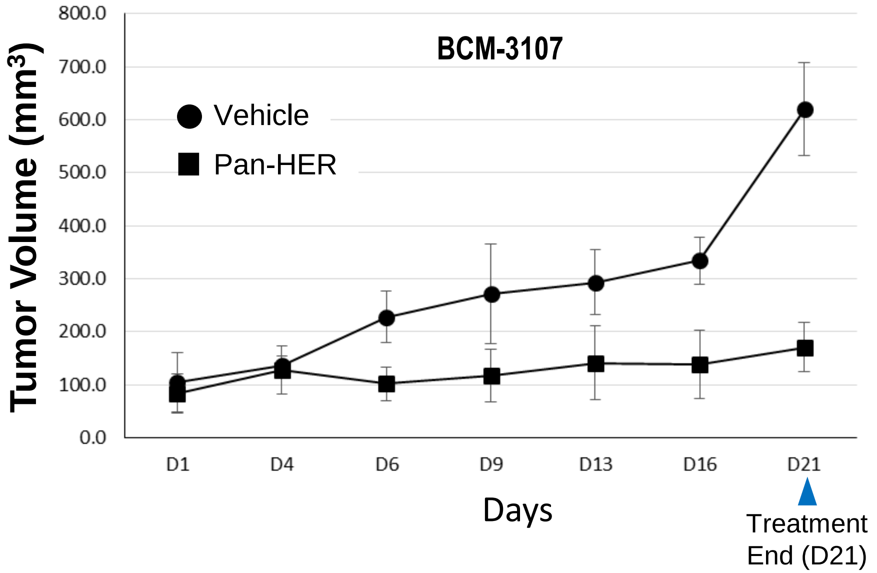
BCM-3107
Vehicle
Pan-HER
Tumor Volume (mm3)
800.0
700.0
600.0
500.0
400.0
300.0
200.0
100.0
0.0
D1
D4
D6
D9
D13
D16
D21
Days
Treatment
End (D21)

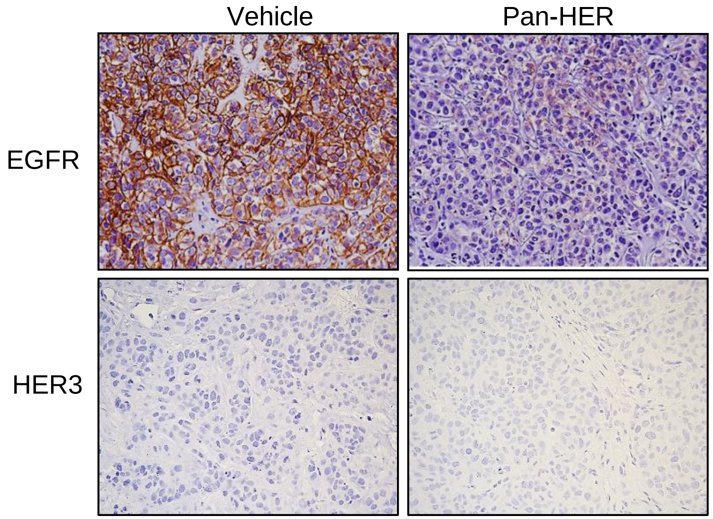
Vehicle
Pan-HER
EGFR
HER3

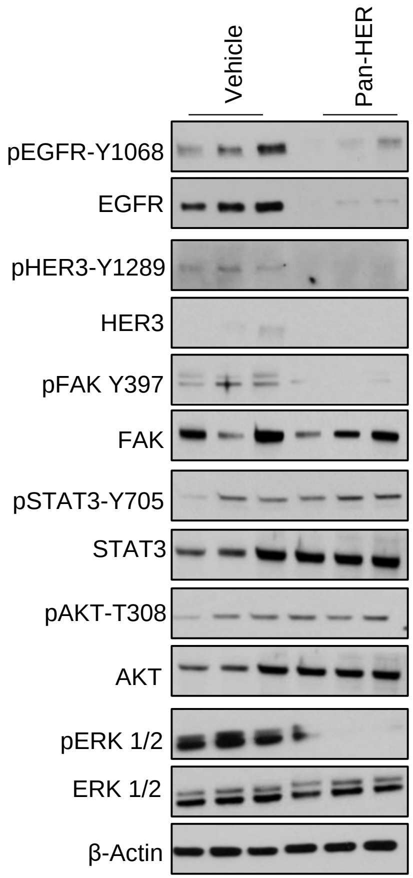
Vehicle
Pan-HER
pEGFR-Y1068
EGFR
pHER3-Y1289
HER3
pFAK Y397
FAK
pSTAT3-Y705
STAT3
pAKT-T308
AKT
pERK 1/2
ERK 1/2
β-Actin

# Figure S12

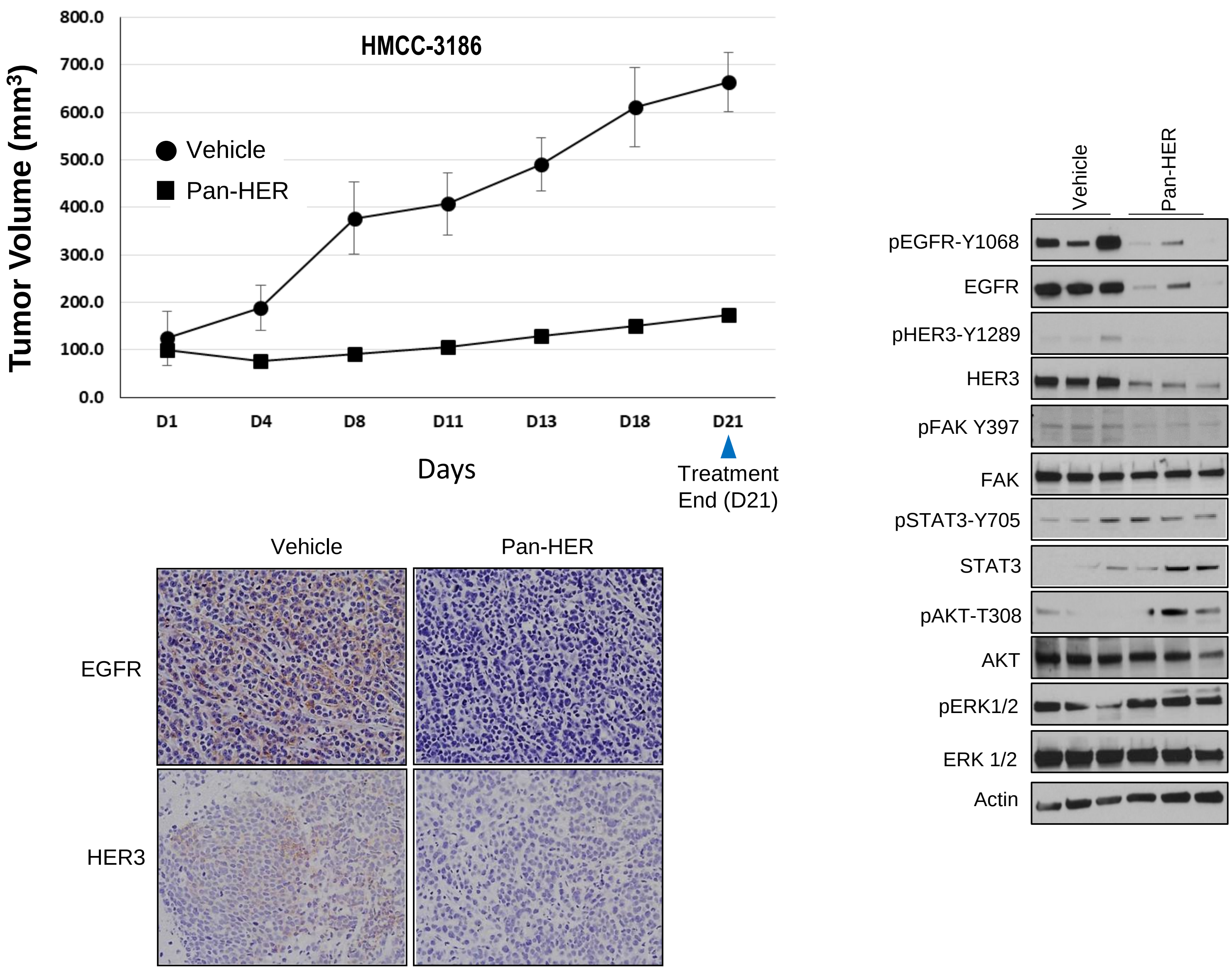
HMCC-3186
Tumor Volume (mm³)
800.0
700.0
600.0
500.0
400.0
300.0
200.0
100.0
0.0
Vehicle
Pan-HER
D1
D4
D8
D11
D13
D18
D21
Days
Treatment End (D21)
Vehicle
Pan-HER
EGFR
HER3
Vehicle
Pan-HER
pEGFR-Y1068
EGFR
pHER3-Y1289
HER3
pFAK Y397
FAK
pSTAT3-Y705
STAT3
pAKT-T308
AKT
pERK1/2
ERK 1/2
Actin

# Figure S13

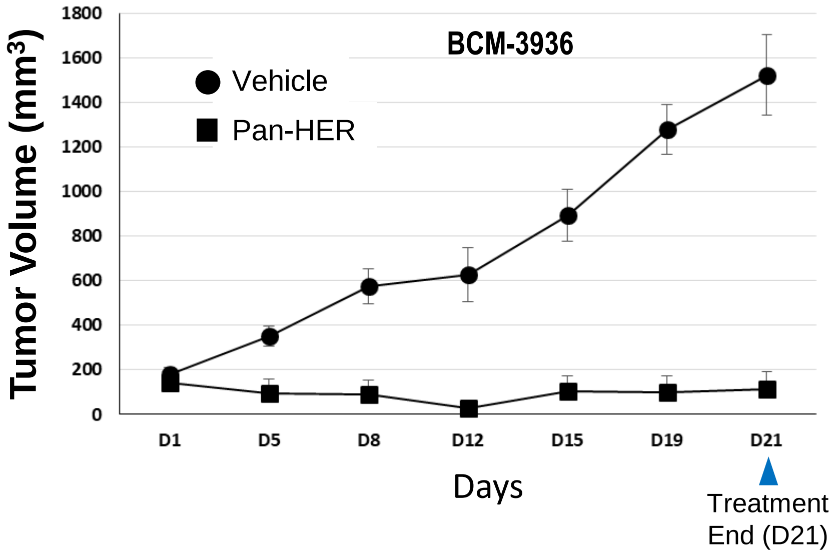

BCM-3936
Vehicle
Pan-HER
Tumor Volume (mm3)
1800
1600
1400
1200
1000
800
600
400
200
0
D1
D5
D8
D12
D15
D19
D21
Days
Treatment End (D21)


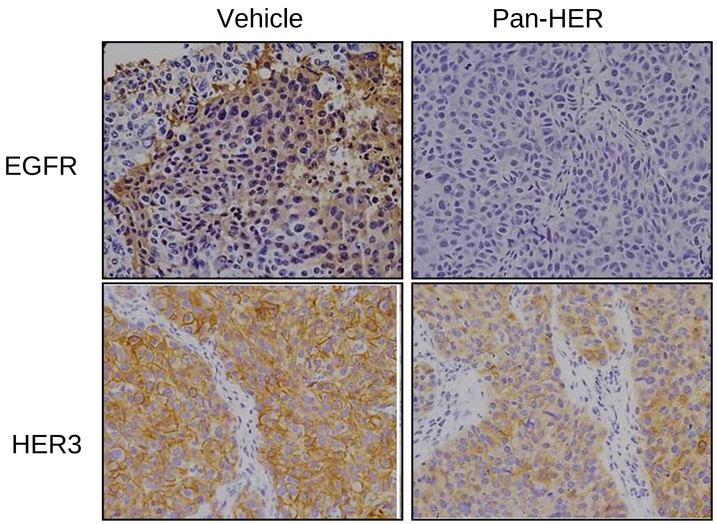

Vehicle
Pan-HER
EGFR
HER3


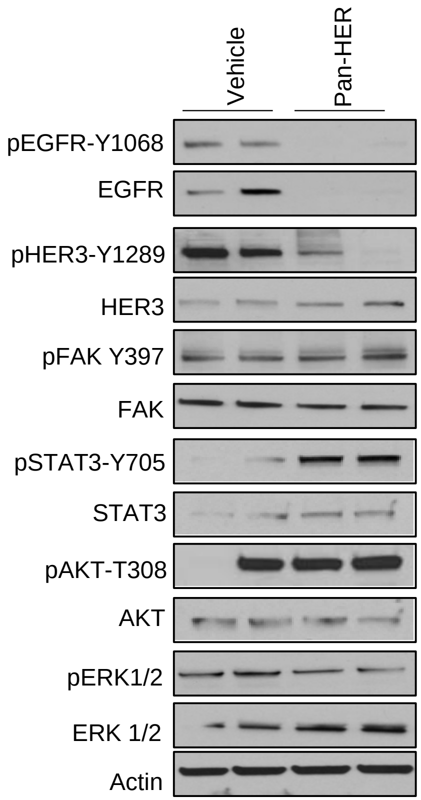

Vehicle
Pan-HER
pEGFR-Y1068
EGFR
pHER3-Y1289
HER3
pFAK Y397
FAK
pSTAT3-Y705
STAT3
pAKT-T308
AKT
pERK1/2
ERK 1/2
Actin

# Figure S14

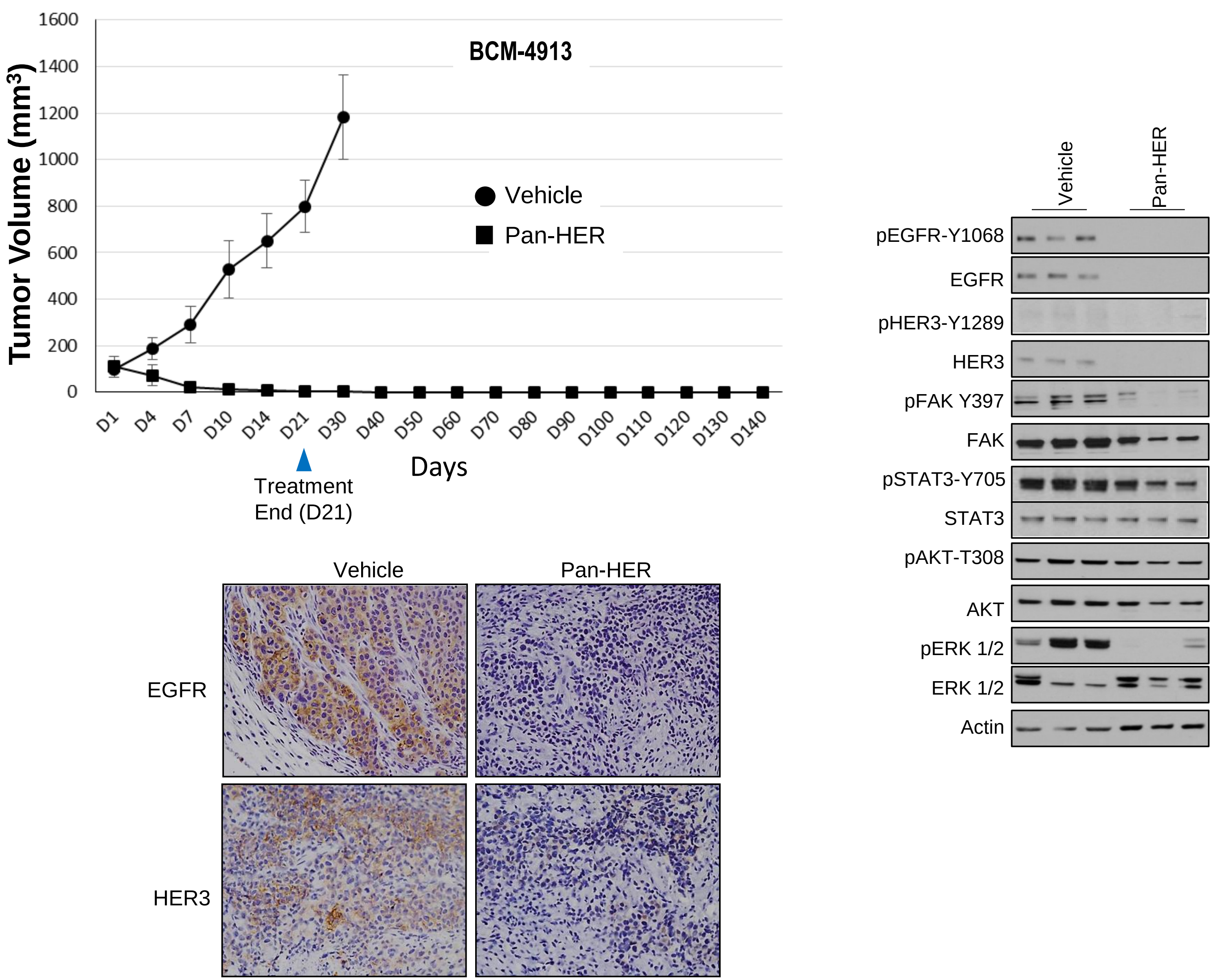
BCM-4913
Tumor Volume (mm³)
1600
1400
1200
1000
800
600
400
200
0
Vehicle
Pan-HER
D1
D4
D7
D10
D14
D21
D30
D40
D50
D60
D70
D80
D90
D100
D110
D120
D130
D140
Days
Treatment
End (D21)
Vehicle
Pan-HER
EGFR
HER3
Vehicle
Pan-HER
pEGFR-Y1068
EGFR
pHER3-Y1289
HER3
pFAK Y397
FAK
pSTAT3-Y705
STAT3
pAKT-T308
AKT
pERK 1/2
ERK 1/2
Actin

Figure S15

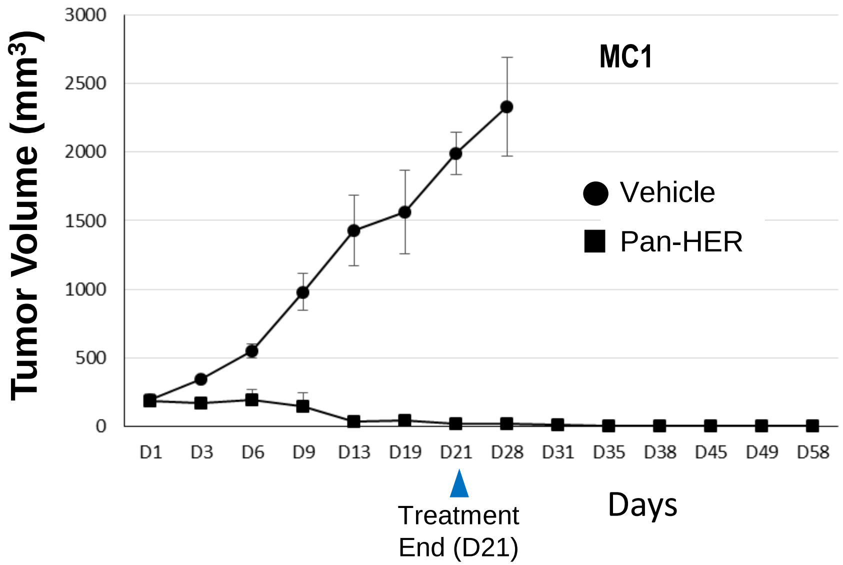

Tumor Volume (mm³)
3000
2500
2000
1500
1000
500
0
MC1
Vehicle
Pan-HER
D1
D3
D6
D9
D13
D19
D21
D28
D31
D35
D38
D45
D49
D58
Treatment
End (D21)
Days


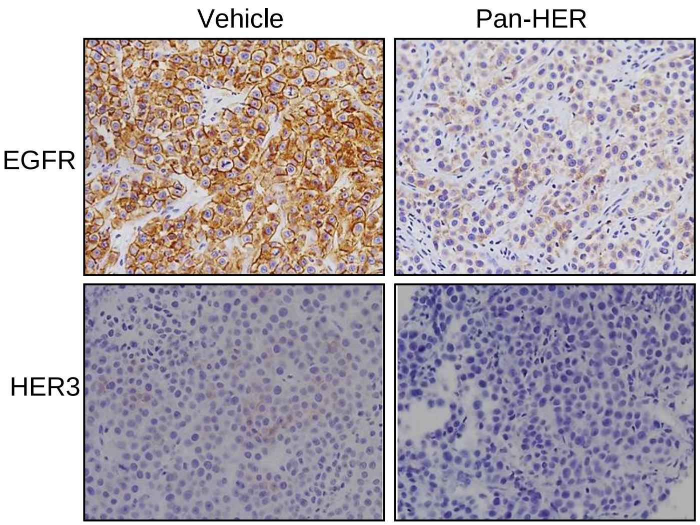

Vehicle
Pan-HER
EGFR
HER3


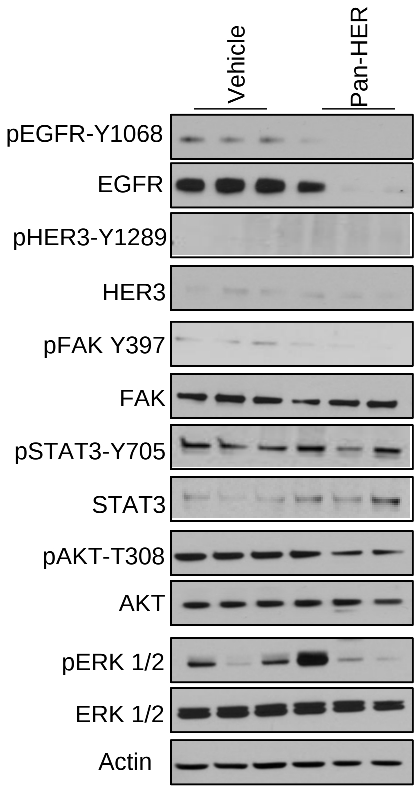

Vehicle
Pan-HER
pEGFR-Y1068
EGFR
pHER3-Y1289
HER3
pFAK Y397
FAK
pSTAT3-Y705
STAT3
pAKT-T308
AKT
pERK 1/2
ERK 1/2
Actin

Figure S16

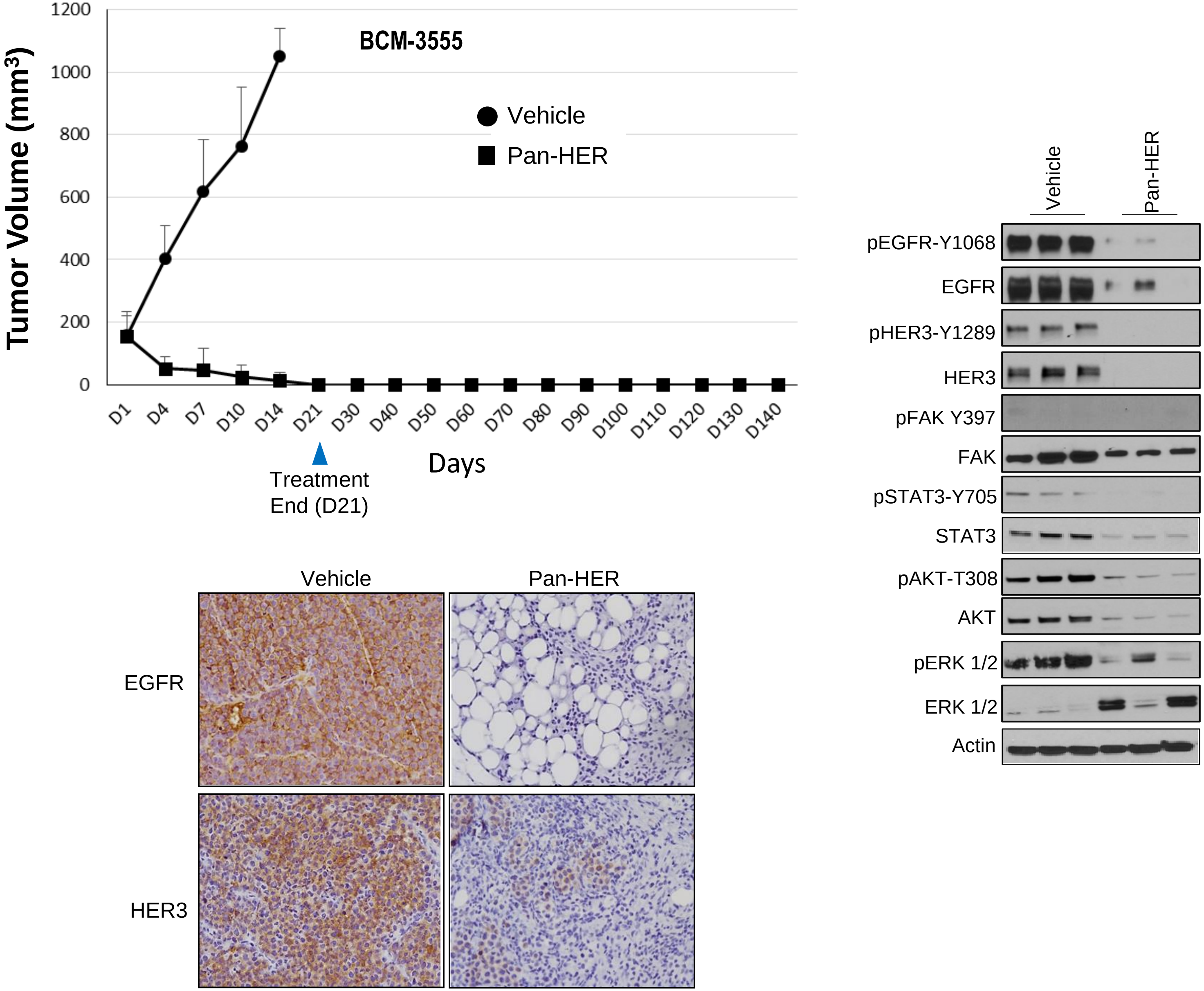
BCM-3555
Tumor Volume (mm³)
1200
1000
800
600
400
200
0
Vehicle
Pan-HER
D1
D4
D7
D10
D14
D21
D30
D40
D50
D60
D70
D80
D90
D100
D110
D120
D130
D140
Days
Treatment End (D21)
Vehicle
Pan-HER
EGFR
HER3
pEGFR-Y1068
EGFR
pHER3-Y1289
HER3
pFAK Y397
FAK
pSTAT3-Y705
STAT3
pAKT-T308
AKT
pERK 1/2
ERK 1/2
Actin

EGF Signaling

**BCM-2147**

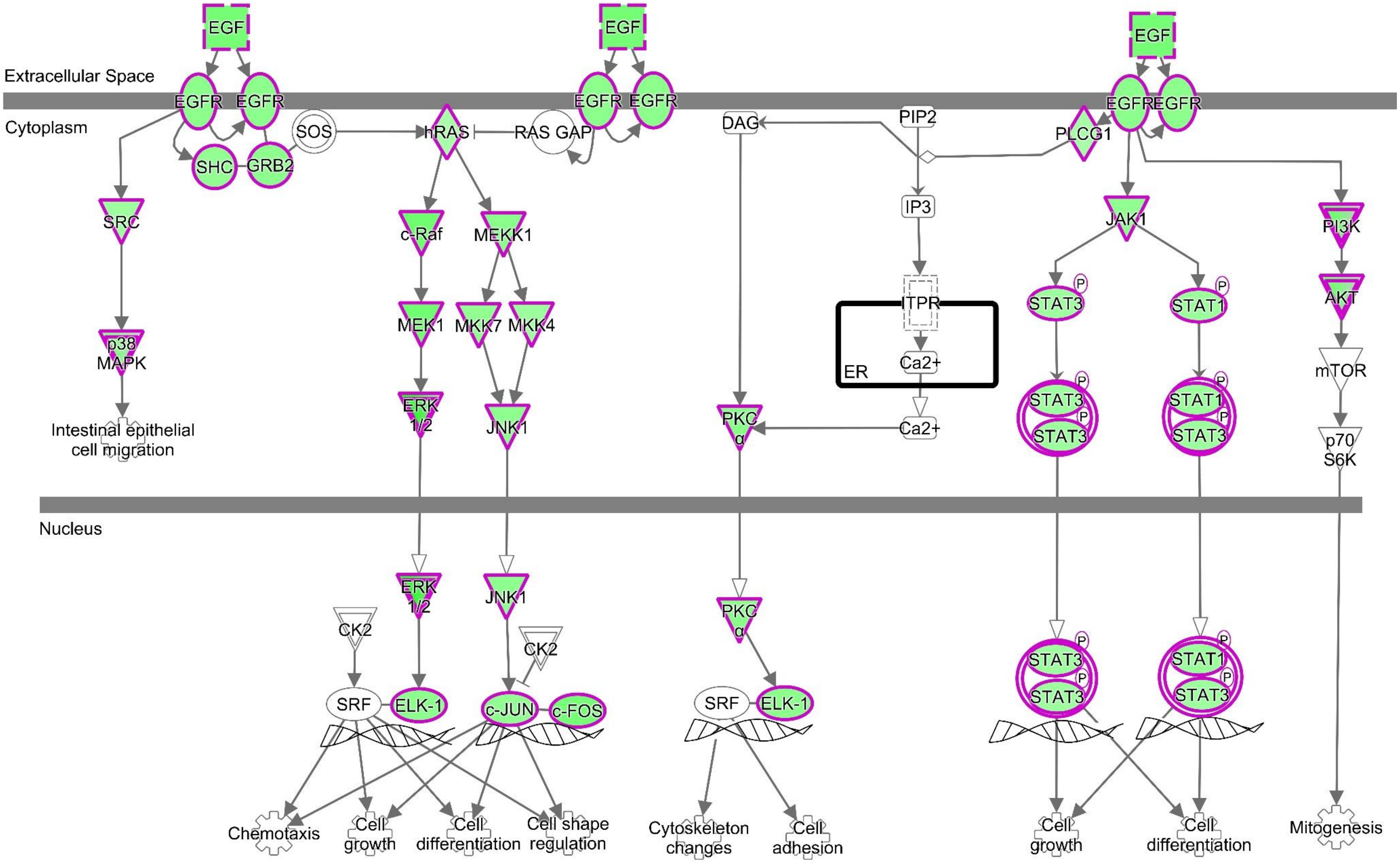

Figure S17.B

NF-κB Signaling

**BCM-2147**

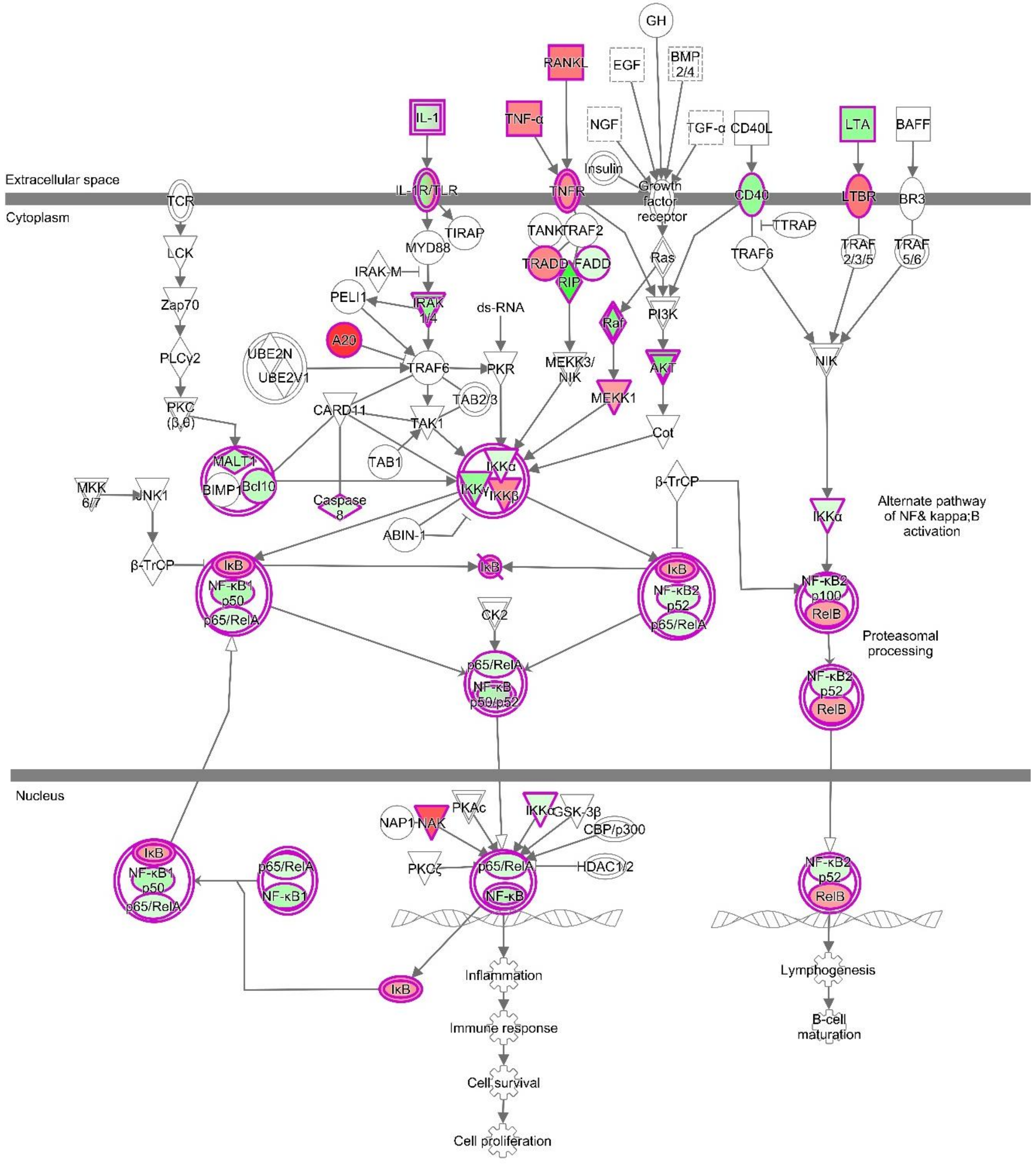

EGF Signaling

**BCM-2665**

Figure S18.A

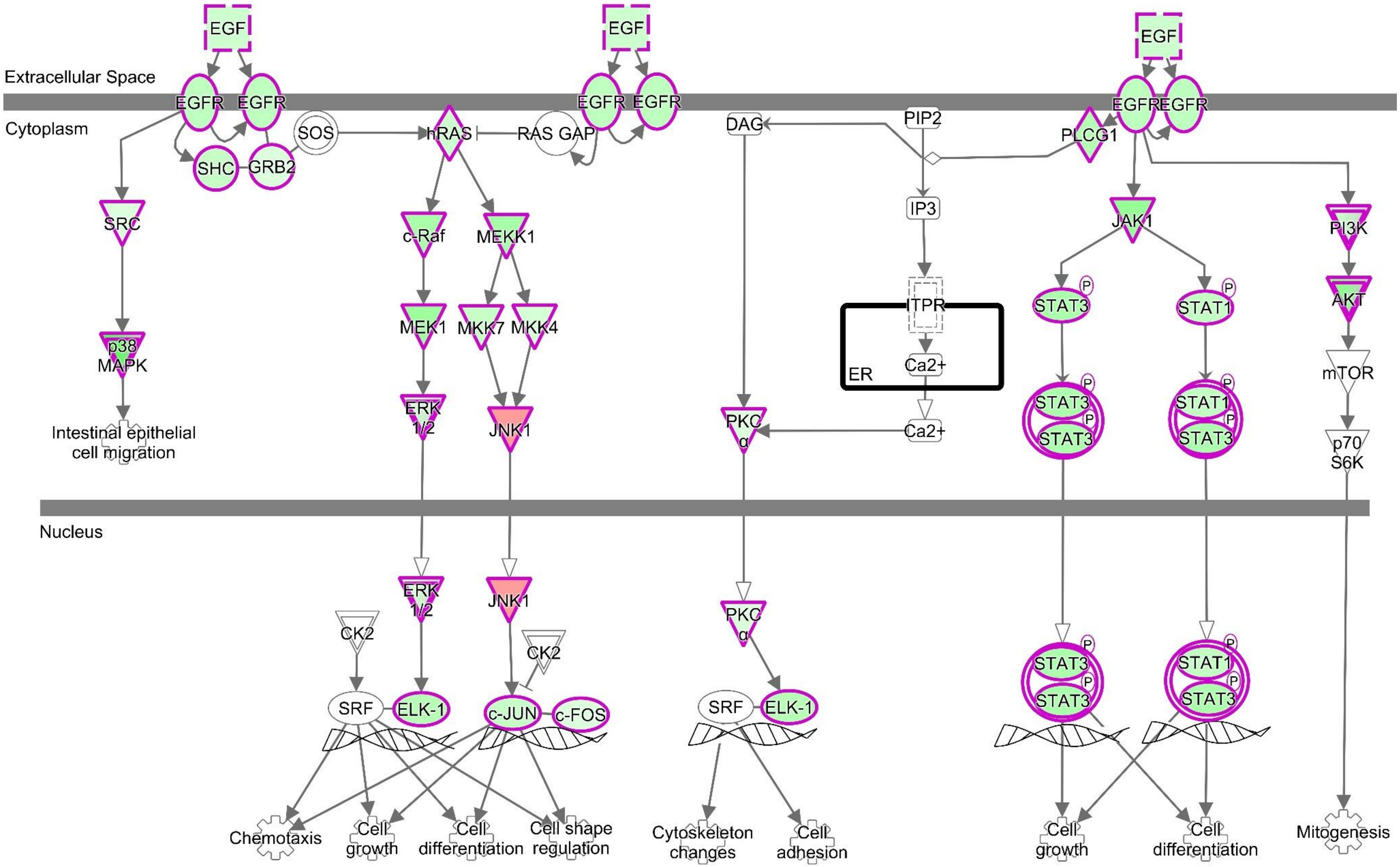

NF-κB Signaling

**BCM-2665**

Figure S18.B

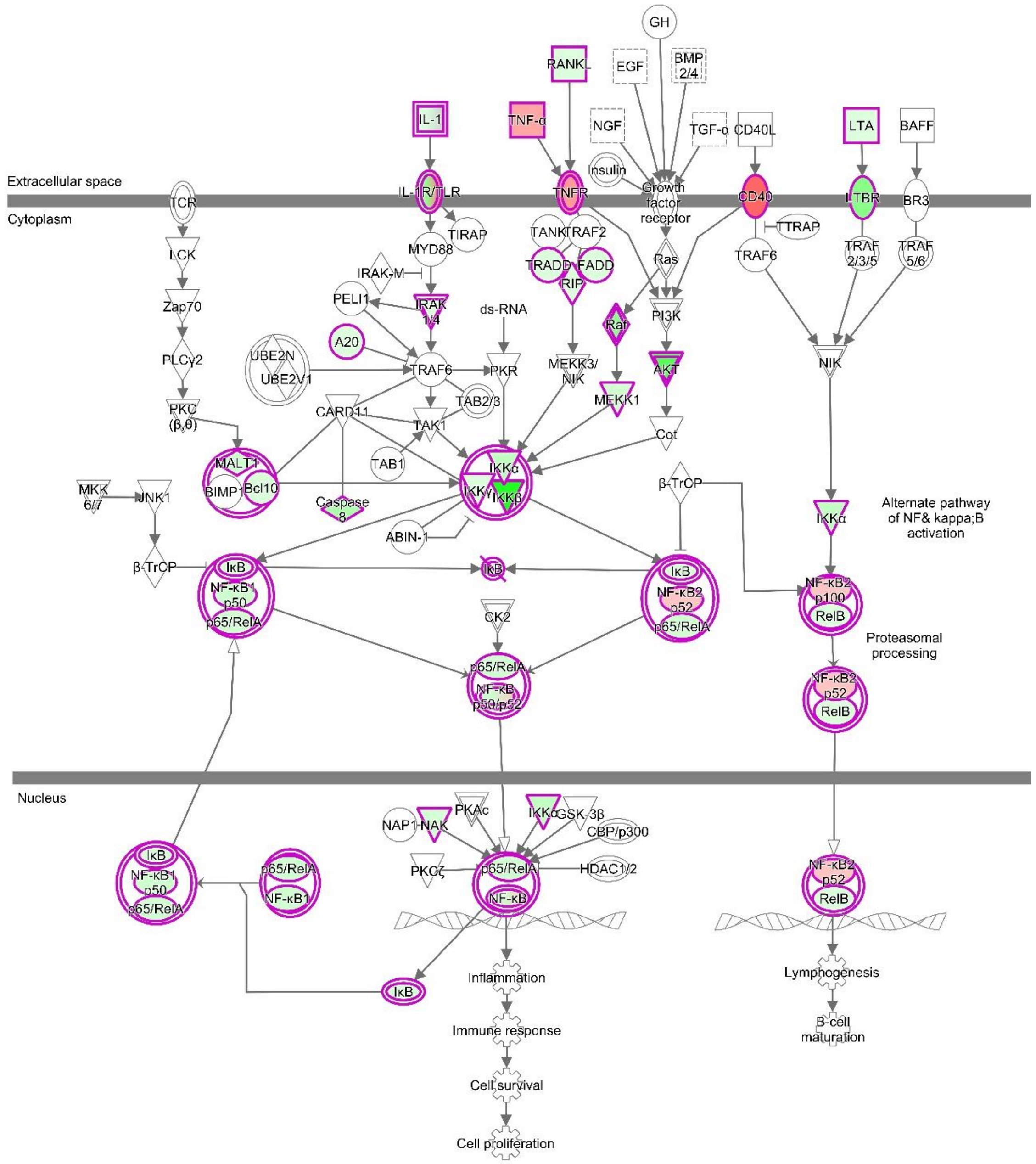

Figure S19.A

EGF Signaling

**BCM-3555**

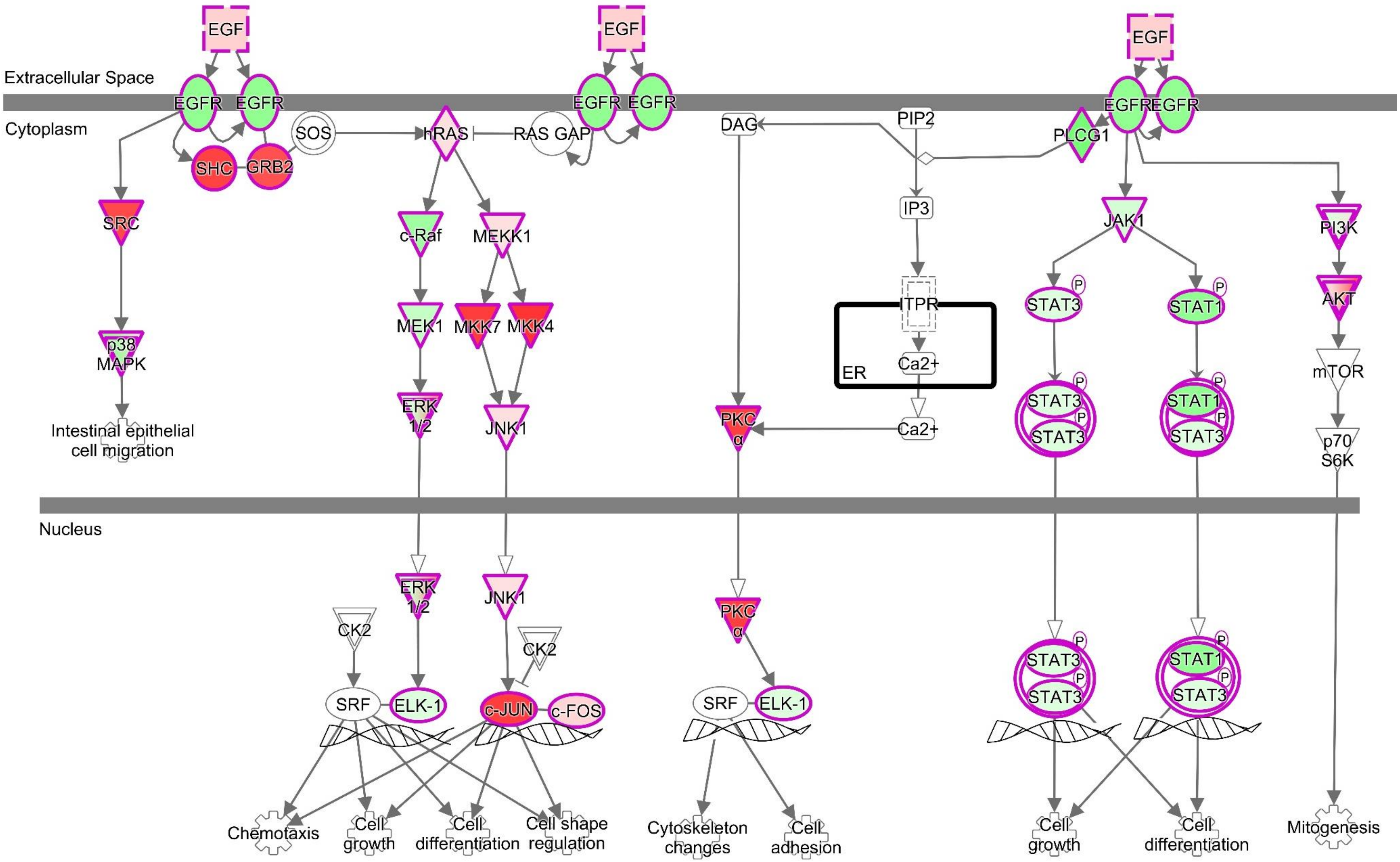

Figure S19.B

NF-κB Signaling

**BCM-3555**

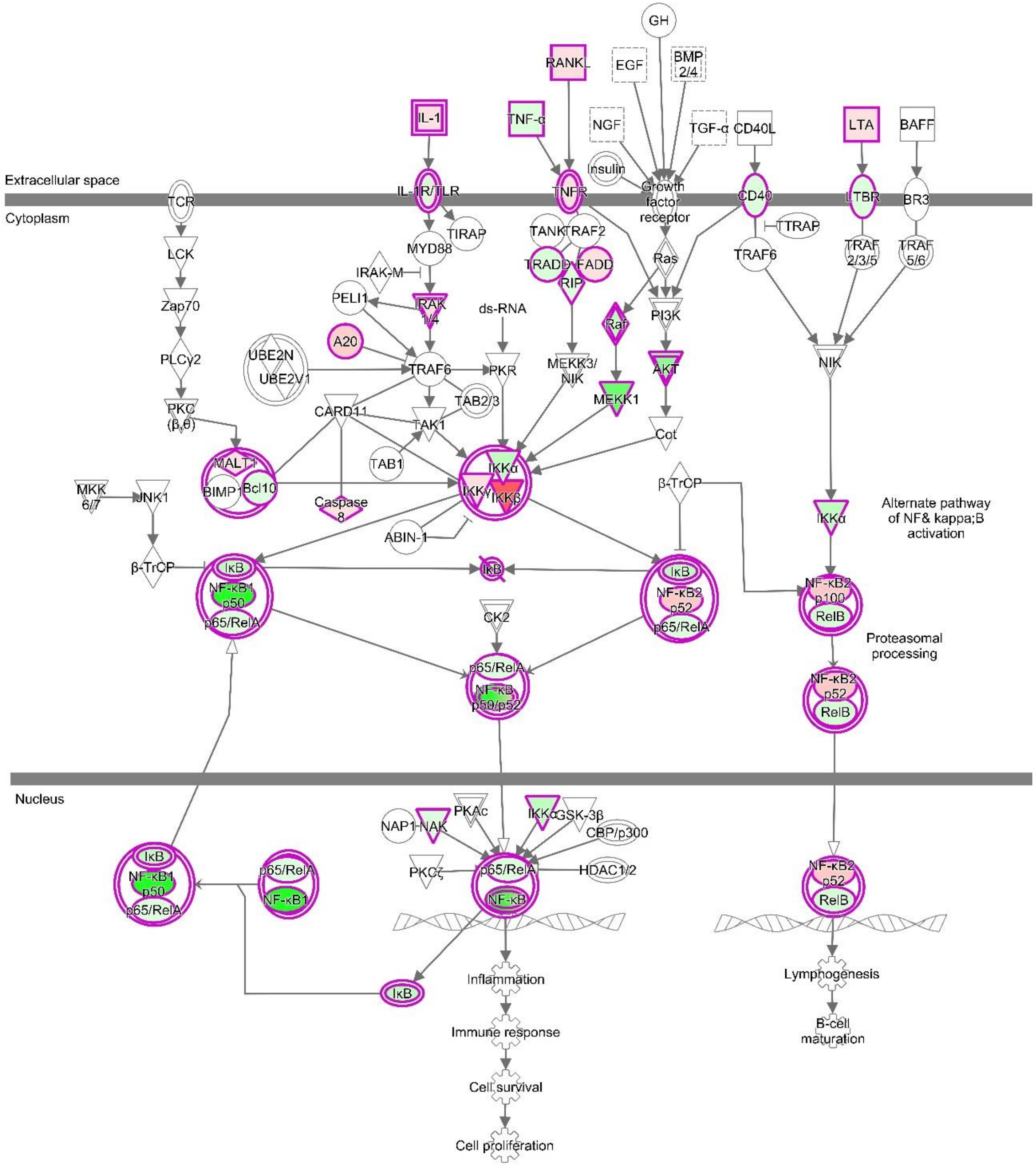

EGF Signaling

**BCM-4913**

Figure S20.A

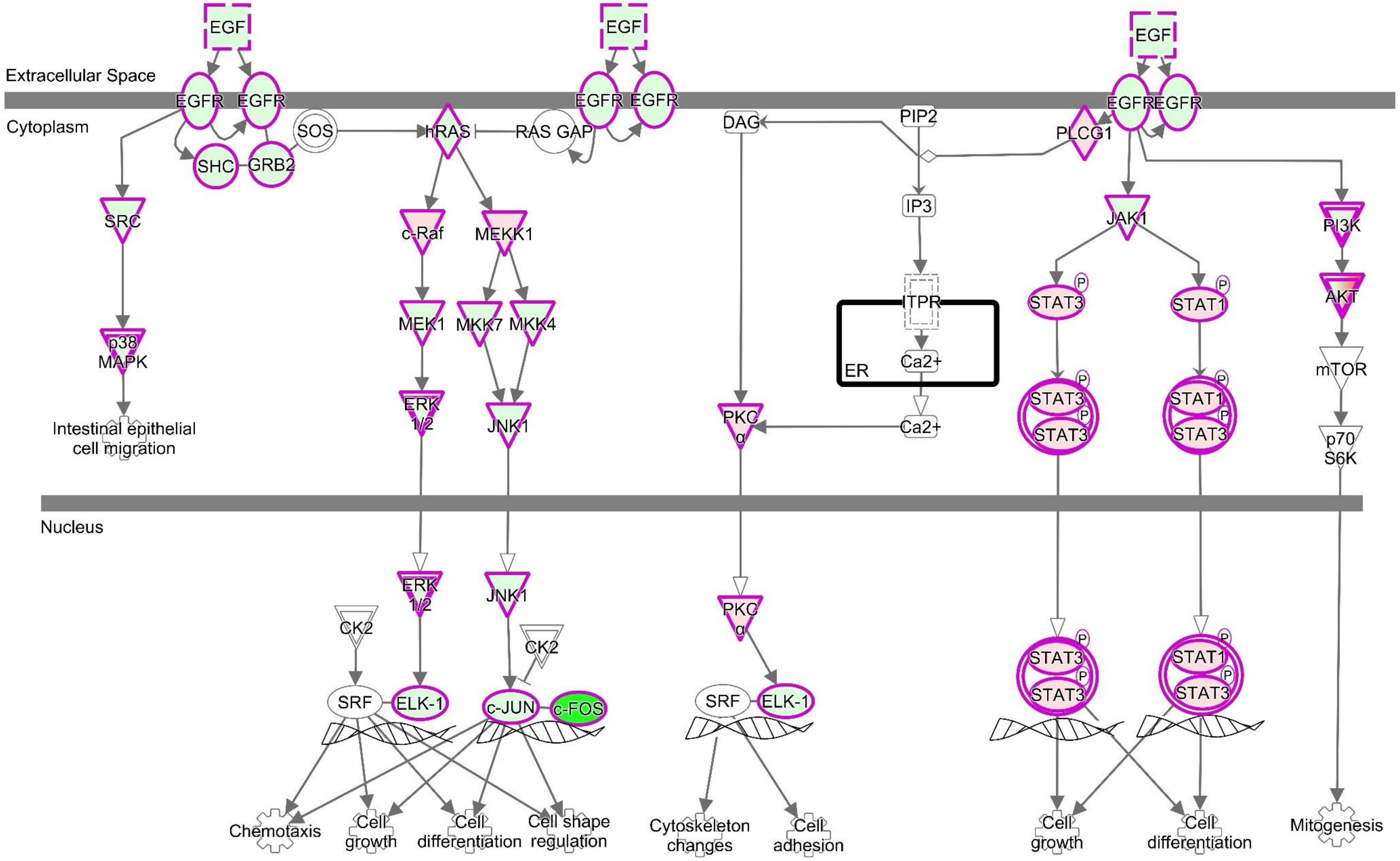

NF-κB Signaling

**BCM-4913**

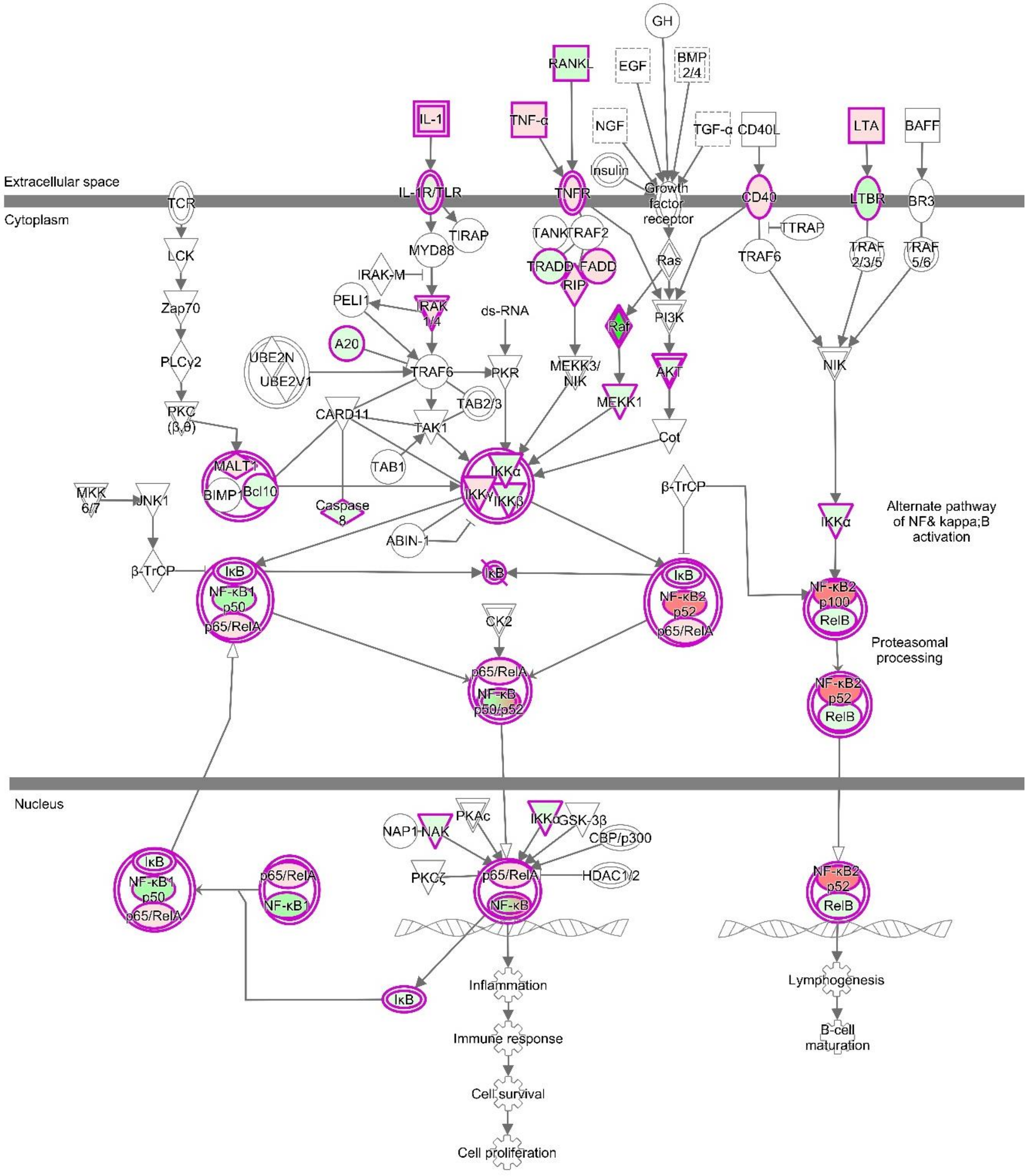

Supplement: Supplementary file 2 — Additional file 2 Figure 1. A, Microarray analysis of RNA gene expression corresponding to PDX samples before treatment. Note the differences in expression of PDXs having shown tumor regression (i.e., BCM-3936, BCM-4913 and MC1; Subgroup 1) vs. the rest of the PDXs (Subgroup 2); PDX BCM-4195 does not express EGFR, HER2, or HER3 and was added to the analysis for comparison only. B, gene expression analysis by Ingenuity Pathway Analysis (Qiagen) comparing BCM-3936, BCM-4913 and MC1 (subgroup 1) vs. the remaining PDXs; it shows among the top molecules a marked reduction of genes related to the AKT/PKB survival pathway including the PTEN pathway, and increased expression of PTK2 (FAK). Supplemental Figures 2-16. Time course analysis of the therapeutic response corresponding to each of the 15 TNBC PDXs used in the present study. A, graph displaying the time-course analysis of tumor growth; B, Western blot analysis of HER family members and associated signaling events; and C, IHC of EGFR and HER3 proteins. Low passage TNBC PDX tumor samples (2 mm × 2 mm) were transferred into the right mammary fat pad of mice for engraftment. Once tumors reached an average size of 150-200 mm3, mice were randomized (n ≥ 3 per group) and treated following the three, one-week cycles design, consisting of 3 times/week IP injection of either formulation buffer (Vehicle control) or Pan-HER (50 mg/kg). Mouse weight was recorded and tumor volumes were measured and calculated as described in Materials & Methods twice weekly. Tumor volume fold change was calculated based on the baseline tumor volumes for each arm. Two-way ANOVA was used for a statistical analysis. At the end of the 3-cycle treatment, the animals were sacrificed and tumors collected for further Western blot and IHC analyses. Supplemental Figures 17-20. EGFR (A) and NF-κB (B) pathway-focused RT-PCR gene expression analysis of representative TNBC PDXs RNA samples collected before and after Pan-HER treatment. RNA samples corresponding [file 13058_2020_1280_MOESM2_ESM.pdf]
