# Supplementary material for: Simultaneous targeting of HER family pro-survival signaling with Pan-HER antibody mixture is highly effective in TNBC: a preclinical trial with PDXs
Source: Breast Cancer Res. 2020 May 15;22:48. doi: 10.1186/s13058-020-01280-z (PMC7227035; doi:10.1186/s13058-020-01280-z)
Supplement: Supplementary file 3 — Additional file 3. DNA extraction and Sanger sequencing of PIK3CA and EGFR exons [file 13058_2020_1280_MOESM3_ESM.pdf]

DNA extraction and Sanger sequencing of PIK3CA and EGFR exons

DNA was extracted from tissue samples of all 15 untreated PDX models using PureLink^{TM} Genomic DNA Mini Kit (Invitrogen – Thermo Fisher, Carlsbad, CA), as specified by the manufacturer. Briefly, 180 µL of Purelink® Genomic Digestion Buffer and 20µL Proteinase K was added to tubes containing ~25 mg tissue sample. Tubes were incubated at 55°C with occasional vortex until lysis was completed. After lysis completion, 20µL RNase was added to lysate, mixed well with vortex, and incubated at room temperature for 2 minutes. 200 µL PureLink® Genomic Lysis/Binding Buffer was added followed by 200 µL 96–100% ethanol to the lysate, and samples were vortexed for five seconds. The lysate mixture was added to PureLink® Spin Column and samples were spun at 10,000 x *g* for 1 minute at room temperature. After centrifugation, collection tube was discarded and was replaced with a new tube. Samples were washed using 500 µL Wash Buffer 1, spun at 10,000 x *g* for 1 minute, collection tube was discarded and replaced with new tube. The same steps described was performed again using Wash Buffer 2. The spin column was placed in a new 1.5 mL microcentrifuge tube and 50-150 µL elution buffer was added to each sample, tubes were incubated at room temperature, and then spun at maximum speed for 1 minute. Upon extraction, total DNA was quantified, aliquoted, and stored in -80°C for later use.

Primers for exons 9 and 20 of PIK3CA and exons 18-21 of EGFR were designed using National Center for Biotechnology Information Human PIK3CA and EGFR reference sequences (NM_006218.3 and NM_005228.5 respectively) and primer-BLAST program. The primers were synthesized at Millipore Sigma (St. Louis, MO), purified, and diluted to 10 µM in molecular grade

water prior to use. Each PCR reaction totaled 25 µL and consisted of 12.5 µL DreamTaq™ Green PCR Master Mix (2X, Thermo Fisher Scientific, Waltham, MA), 1.25 µL forward primer, 1.25 µL forward primer, 25 ng template DNA, and sufficient volume of nuclease-free water to reach 25 µL total volume. Reactions were heated to 95°C for 2 minutes and then subjected to 35 cycles of PCR using the following parameters: 30-second denaturation at 95°C, followed by 30-sec annealing at 61.8°C, followed by 1-minute extension at 72ºC. After the 35th cycle, the reactions were incubated for 10 minutes at 72°C and then held at 4°C.

PCR products were analyzed by performing DNA gel electrophoresis on a 2% agarose gel stained with ethidium bromide. PCR samples containing PI3KCA and EGFR-specific amplicons were loaded onto agarose gel and ran at 80V for 1 hour. Gels were visualized under ultraviolet light and bands specific to amplified product were excised. Amplicons were purified using GeneJET Gel Extraction Kit (Thermo Scientific). Briefly, a 1:1 volume of binding buffer from the gel extraction kit (based on volume:weight) was added to each tube containing excised gel. The gel mixture was incubated at 55°C for 10 minute or until gel completely dissolved. 400 µL of isopropanol was added to each tube and the solubilized gel solution was transferred to a GeneJet purification column. The tubes were centrifuged at maximum speed for 1 minute and flow through was discarded. 100 µL of binding buffer was added to each spin column, tubes were spun at maximum speed for 1 minute and flow through was discarded. 700 µL of wash buffer was added to each spin column, tubes were spun at maximum speed for 1 minute and flow through was discarded. The spin columns were then transferred into clean 1.5 mL microcentrifuge tubes, 50 µL elution buffer was added to center of purification column membrane and columns were spun at maximum speed for 1 minute. The purified amplicon samples were quantified and 10 ng of each

purified sample was prepared for Sanger sequencing. Sanger sequencing samples were sent to GeneWiz (South Plainfield, NJ) for processing. Sequences were analyzed using PIK3CA and EGFR NCBI reference sequences NM_006218.3 and NM_005228.5, respectively, and Multiple Sequence Comparison by Log-Expectation (MUSCLE) program.

Table S4: Primers used to amplify the exons corresponding to PI3KCA and EGFR genes used to analyze the presence of mutations and/or genetic alterations.

PIK3CA		
Exon 9	Forward	5’-GAGTAACAGACTAGCTAGAGAC-3’
	Reverse	5’-CTGTGACTCCATAGAAAATCTTC-3’
Exon 20	Forward	5’-GTTTCAGGAGATGTGTTACAAGGC-3’
	Reverse	5’-CAGTTCAATGCATGCTGTTTAATTGTG-3

EGFR		
Exon 18	Forward	5’-AACGTTGCCTTAGAAGCCTGT-3’
	Reverse	5’-GGTGCAGCCTAGAAACATTGC-3’
Exon 19	Forward	5’-AGAACACCTGTATCAGAGCCT-3’
	Reverse	5’-TCCTCTCAATAACTTGGGAAAAAC-3’
Exon 20	Forward	5’-TCAGTGTGATTCGTGGAGCC-3’
	Reverse	5’-GTGCTGGGTAGATGCCAGTA-3’
Exon 21	Forward	5’-GTCCCTGTGCTAGGTCTTTTG-3’
	Reverse	5’-CACACATATCCCCATGGCAAAC-3’
